# Naturally occurring hybrids derived from γ-amino acids and sugars with potential *tail to tail* ether-bonds

**DOI:** 10.1038/srep25443

**Published:** 2016-05-11

**Authors:** Zi-ming Feng, Zhi-lai Zhan, Ya-nan Yang, Jian-shuang Jiang, Pei-cheng Zhang

**Affiliations:** 1 State Key Laboratory of Bioactive Substance and Function of Natural Medicines, Institute of Materia Medica, Chinese Academy of Medical Sciences and Peking Union Medical College, Beijing 100050, P. R. China; 2 State Key Laboratory Breeding Base of Dao-di Herbs, National Resource Center for Chinese Materia Medica, China Academy of Chinese Medical Sciences, Beijing, 100700, P. R. China

## Abstract

The basic substances of life include various amino acids and sugars. To search such molecules is the precondition to understand the essential nature. Here we reported four unprecedented hybrids of *γ*-amino acids and sugars from the roots of *Ranunculus ternatus*, which possess potential *tail to tail* ether-connected (6,6-ether-connected) modes in the sugar moiety. The structures of these hybrids were elucidated by extensive analyses of spectra and calculated electronic circular dichroism (ECD) method.

Amino acids and sugars perform vital roles in all living organisms. They are involved in complex biological processes such as catalysis and highly selective molecular recognition. Their hybrids, sugar amino acids (SAAs), which feature both a sugar framework and amino acid functional groups, have attracted substantial interest for their roles in peptidomimetic studies^1^,^2^. Herein, we report four novel hybrids of amino acids and sugars, ranunculins A-D (**1**–**4**), from *Ranunculus ternatus* Thunb, a plant used in traditional Chinese Medicine. The plant is an annual herb belonging to the Ranunculaceae family, and its root is used in traditional Chinese Medicine as a treatment for faucitis. Additionally, the ethanol extract of the plant has clearly exhibited antitumor and antituberculosis activities^3^. Previous investigations of this plant have revealed the presence of triterpenes, biflavonoids, indolopyridoquinazoline alkaloidal glycosides, and other glycosides^3^,^4^. As part of the ongoing study of the structural diversity of natural compounds, we focused on the water-soluted fraction of the ethanol extract of *R. ternatus*, which other researchers have not exploited, four novel compounds (Fig. 1) were isolated. Different from normal SAAs and aminoglycosides^5^,^6^,^7^, which contain aminosugar substructures, **1**–**4** possess a relative integrated *γ*-aminobutyric acid (GABA) moiety and monosaccharide or disaccharide moieties. More intriguingly, all of the compounds have potential *tail to tail* ether-connected (6,6-ether-connected) bonds. This represents an unprecedented structural phenomenon for the connections of sugars with the participation of *γ*-amino acids. Considering the significant roles of sugars and *γ*-amino acids, these novel compounds could provide opportunities for expanding our understanding of various natural chemical and biosynthetic phenomena.

## Results

**Figure 2:**
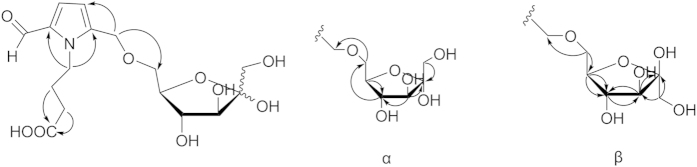
Selected HMBC correlations of compound 1.

Compound **1** was obtained as a yellow amorphous powder. Its molecular formula was determined to be C_16_H_23_NO_9_ based on the high resolution electrospray ionization mass spectroscopy (HRESIMS) quasi-molecular ion at *m*/*z* 396.1266 [*M* + Na]^+^ (calcd for C_16_H_23_NO_9_Na, 396.1265), corresponding to 6 degrees of unsaturation. The infrared radiation (IR) spectrum of **1** revealed the presence of hydroxyl (3388 cm^−1^), carbonyl (1712 cm^−1^), and aldehyde (1649 cm^−1^) groups. The ^1^H NMR spectrum (Table 1) of **1** displayed an aldehyde group at *δ* 9.48 (1H, s, CHO), a system of *sp*^2^ aromatic resonances at *δ* 6.98 (1H, d, *J* = 3.5 Hz) and 6.27 (1H, d, *J* = 3.5 Hz), and four methylenes at *δ* 4.50 (2H, s), 4.25 (2H, t, *J* = 7.5 Hz), 2.20 (2H, t, *J* = 7.5 Hz), and 1.85 (2H, m, *J* = 7.5 Hz). Interestingly, instead of one remaining sugar moiety, which would be expected considering the molecular weight of the compound, two sets of similar sugar resonances were observed; additionally, the ^13^C NMR sugar resonances were presented in pairs, implying that two anomers of the sugar were in equilibrium. In the ^13^C NMR spectrum (Table 1), the resonances showed two sets of fructose carbon signals. Moreover, the aldehyde group at *δ* 179.4, two carbonyl signals at *δ*173.9 and 173.8, six *sp*^*2*^ carbon signals at *δ*139.2, 139.1, 132.0, 123.6, 111.2, and 111.1, two oxygenated methylene carbons at *δ* 63.6 and 63.3, and three methylenes at *δ* 44.2, 30.7, and 26.3 were observed. Furthermore, the resonances at *δ*_C_132.0, 123.6, 111.2, 139.2, and 179.4 suggested the presence of a pyrrole-2-aldehyde skeleton. In the heteronuclear multiple bond correlation (HMBC) experiment (Fig. 2), the proton H-4 (*δ* 4.25) correlated with C-2′ (*δ* 132.0), C-5′ (*δ* 139.2), C-3 (*δ* 26.3) and C-2 (*δ* 30.7); H-3 (*δ* 1.85) correlated with C-4 (*δ* 44.2), C-2, and C-1 (*δ* 173.9); H-2 (*δ* 2.20) correlated with C-1 and C-3, indicating the existence of a 4-(2-formyl-1H-pyrrol-1-yl) butanoic acid moiety. Furthermore, an oxygenated methylene hydrogen signal at H-6′ (*δ* 4.50) correlated with C-4′ (*δ* 111.2) on the pyrrol ring, as well as C-6″α (*δ* 70.8) and C-6″β (*δ* 72.4) on the fructose moiety. Additionally, correlations between H-6″β (*δ* 3.57, 3.45) and C-6′β (*δ* 63.3), and H-6″α (*δ* 3.44, 3.43) and C-6′α (*δ* 63.6) were observed, which indicated that the oxygenated methylene of the 4-(2-formyl-1H-pyrrol-1-yl) butanoic acid moiety was connected with position C-6 on the fructose moiety through an ether linkage. Thus, compound **1** was determined and named ranunculin A.

**Figure 3:**
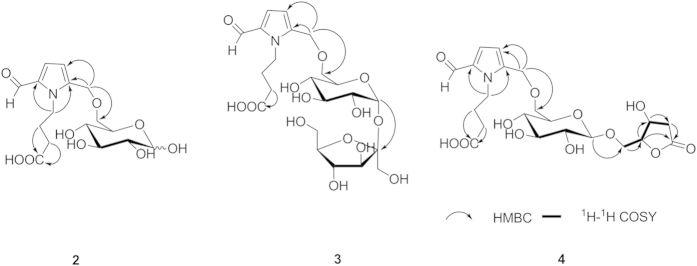
Selected correlations of compounds 2–4.

Compound **2** was also obtained as a yellow amorphous powder. The HRESIMS gave a quasi-molecular ion at *m/z* 396.1266 [*M* + Na]^+^ (calcd for 396.1265), which corresponded to the molecular formula C_16_H_23_NO_9_. The IR, UV, and NMR data (Table 1) of **2** were similar to the corresponding data of **1**; both sets of data showed the presence of a 4-(2-formyl-1*H*-pyrrol-1-yl) butanoic acid moiety. However, NMR resonances for a fructose moiety in **1** were absent and were replaced by signals attributed to the glucose moiety in **2**. Furthermore, correlations between H-6″ and C-6′ (*δ* 63.5 and 63.4) of the 4-(2-formyl-1*H*-pyrrol-1-yl) butanoic acid moiety and H-6′ (*δ* 4.50) and C-6″ (*δ* 70.0, 69.9) of the glucose moiety were observed in the HMBC spectra (Fig. 3). Consequently, compound **2** was determined and named ranunculin B.

The molecular formula of **3** was established as C_22_H_33_NO_14_ by the HRESIMS ion at *m/z* 558.1791 [*M* + Na]^+^ (calcd for 558.1793). Comparisons of the molecular weight and sites of unsaturation of compound **3** with those of **2** suggested that an additional hexose moiety existed in compound **3**. Further analysis of the NMR spectra showed that most of the data were similar, with the exception of a set of fructose signals (Table 2). By contrast, the ^13^C NMR spectroscopic data of compound **3** were not presented in pairs as in **1** and **2**, which suggested that the anomeric proton of the sugar was fixed by other substituted groups. The anomer of the fructose was determined to be *β*-configuration by comparing its NMR data with those of compound **1** (Table 1). In the HMBC experiment (Fig. 3), the correlations between the anomeric proton *δ* H-1″ (*δ* 5.12, *J* = 3.5) of the glucose moiety and C-2″′(*δ* 104.1) of the fructose moiety and H-6′ (*δ* 4.50) and C-6″ (*δ* 71.9) indicated that the oxygenated methylenes of 4-(2-formyl-1*H*- pyrrol-1-yl) butanoic acid moiety were connected with the C-6 position of the glucose moiety through an ether linkage, while the C-1 position of *β*-fructose was connected with the anomeric proton of the glucose moiety through an *α*-linkage. Thus, compound **3** was determined and named ranunculin C.

**Figure 4:**
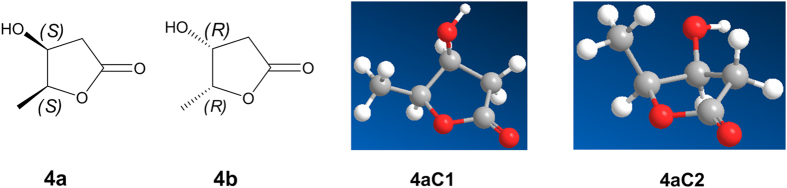
The substructure of 4 and the conformation of 4a.

**Figure 5:**
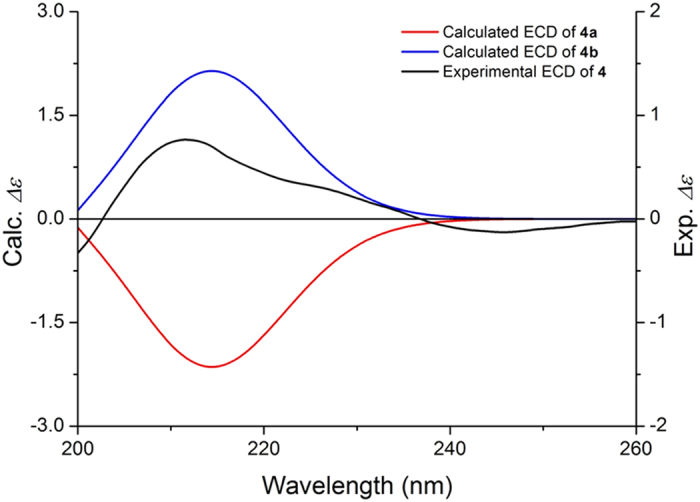
Experimental ECD spectra of 4 and calculated ECD of 4a and 4b in H_2_O.

Compound **4** was obtained as a yellow amorphous powder. An HRESIMS analysis provided an [*M* + Na]^+^ ion at *m/z* 510.1587, appropriate for a molecular formula of C_21_H_29_NO_12_ (calcd for [M + Na]^+^, 510.1582) requiring 8 sites of unsaturation. Based on the NMR information, a substructure similar to that of compound **2** could be readily deduced, considering the aforementioned structural analysis. However, in contrast to the latter, the NMR signals of **4** were not in pairs, which suggested that the anomeric proton of the glucoside was substituted. In addition to these similar resonances, the ^1^H NMR (Table 2) showed two oxygenated methylenes at *δ* 4.00 (1H, m) and 3.68 (1H, dd, *J* = 12.0, 8.0 Hz), two methylenes at *δ* 2.82 (1H, dd, *J* = 17.0, 5.5 Hz) and 2.25 (1H, d, *J* = 17.0 Hz) and two oxygenated methines at 4.52 (1H, m) and 4.39 (1H, m). In the ^13^C NMR spectrum, an ester carbonyl carbon at *δ* 175.9, one methylene carbon at *δ*  38.8, and three oxygenated carbons at *δ* 83.2, 67.6, and 67.1 were observed. The ^1^H-^1^H correlated spectroscopy (^1^H-^1^H COSY) spectrum (Fig. 3) showed correlations between H-1″′ (*δ* 4.00; 3.68) and H-2″′ (*δ* 4.52), between H-2″′ and H-3″′ (*δ* 4.39), and between H-3″′ and H-4″′ (*δ* 2.82; 2.25), suggesting the existence of an additional four-chain carbon moiety. Furthermore, in the HMBC spectra (Fig. 3), the correlations between H-3″′, H-4″′, and H-2″′ and the ester carbon C-5″′ (*δ* 175.9) were observed; thus, **4** should contain a five-membered lactone with a methylene substitute combing the remaining 2 degrees of unsaturation. Moreover, the HMBC correlations between the methylene proton H-1″′(*δ* 4.00 and 3.68) and the glucoside anomeric carbon C-1″ (*δ* 103.0), and between the glucoside anomeric proton H-1″ (*δ* 4.23) and the C-1″′ indicated that the five-membered lactone was connected with an anomeric carbon of the glucoside moiety through a methylene bond and that the *β*-configuration of the glucoside was determined based on the coupling constant, *J* = 8.0, of the anomeric proton. The configurations of C-2″′ and C-3″′ were deduced to be 2″′*S*, 3″′*S* or 2″′*R*, 3″′*R* on the basis of related literature^8^,^9^. Subsequently, their absolute configurations were established by comparisons of the experimental CD spectra and the calculated ECD data. Because too many conformations were produced by the free rotaries of its single bonds while some of them had little effect on the determination of the absolute configuration of C-2″′ and C-3″′, the simplified structures **4a** and **4b** (Fig. 4), were used for ECD calculations. The optimized conformations of **4aC1** (92.00%) were obtained by a systematic conformational analysis, which was performed for **4a** using an MMFF94 molecular mechanics force field calculation (Fig. 4). Using the time-dependent density functional theory (TD-DFT) method at the B3LYP/6-31G(d) level, the overall calculated ECD spectra of **4a** were generated by Boltzmann weighting of their lowest energy conformations. The overall patterns of the calculated ECD spectra of **4b** attributable to 2″′*R*, 3″′*R* were consistent with the experimental data for **4** throughout the entire range of wavelengths under investigation (Fig. 5). Thus, compound **4** was determined and named ranunculin D.

## Discussion

**Figure 6:**
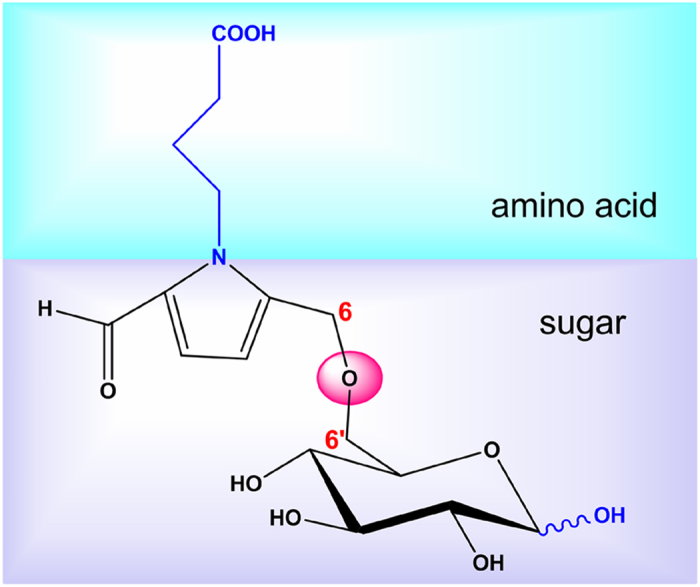
The structural characteristic of compounds 1–4 (using 2 as a representative).

Structurally, for ranunculins A-D (Fig. 6), their potential 6-hydroxymethyls of 3-deoxyglucosones (3-DG) connected with another 6-hydroxymethyl group of sugars through a 6,6′-ether-bond (*tail to tail* ether-bond)^10^,^11^,^12^,^13^,^14^,^15^. In nature, such types of connections are unusual, in contrast to the familiar *head to head* (*e.g.,* trehalose: 1, 1 connected), *head to tail* (*e.g.,* isomaltose: 1, 6 connected) and *head to body* (*e.g.,* sucrose: 1, 2 connected) connections. Coyolosa, a so-called 6,6′-ether-connected sugar, was reported to have been isolated from the root of *Acrocomia Mexicana* in one study^16^. However, very soon thereafter, this structure was regarded to be an incorrect and controversial based on the originally reported NMR spectroscopic data and activity studies^17^,^18^,^19^. Indeed, it is impossible that the ^13^C NMR data of C-6 were still at *δ*_C_ 61.1 when the 6,6′-ether-connection was formed. Nevertheless, inspired by the proposed coyolosa, Takahashi *et al.*^17^ and Haines, A. H.^18^,^19^ developed novel synthesis methods for 6,6′-ether-connected pyranoses. However, to date, no *tail to tail* connected sugars have been found from natural products, not to mention the participation of *γ*-amino acid moieties. Thus, compounds **1**–**4** represent a new connective sample between a 6,6′-ether-connected sugar and *γ*-amino acid. Subsequently, different synthetic routes were designed to obtain such compounds. For example, the reactions between 4-(2-formyl-5-hydroxymethyl-1*H*-pyrrol-1-yl) butanoic acid esters and 6-iodo- or 6-trifluoromethanesulfonate- substituted sugars with the remaining hydroxyls by acetonide protection or between 4-(2-formyl-5-chloro-1*H*-pyrrol-1-yl) butanoic acid esters and the aforementioned sugars with the remaining hydroxyls by acetonide protection, among others. Unfortunately, all of these attempts failed. Thus, these unusual molecules could challenge the synthetic skills of curious investigators. For living organisms in nature, *γ*-aminobutyric acid is the major inhibitory neurotransmitter in the mammalian central nervous system, whereas D-glucose is one of the main products of photosynthesis and fuel sources for cellular respiration. What factors will play a role in the sustainability of such molecules when they form conjugates by such connection modes? Perhaps only when large amounts of synthetic products are obtained will this exploration be successful.

## Methods

### General experimental procedures

The IR spectra were obtained using an IMPACT 400 (KBr) spectrometer. ^1^H NMR (500 MHz), ^13^C NMR (125 MHz), and 2D-NMR spectra were recorded with an INOVA-500 spectrometer with TMS as an internal standard. Values were given in ppm. HRESIMS were obtained using an Agilent 1100 series LC/MSD ion trap mass spectrometer. The optical rotations were measured with a Jasco P-2000 polarimeter. Column chromatography was performed on macroporous resin (Diaion HP-20, Mitsubishi Chemical Corp., Tokyo, Japan), Rp-18 (50 μm, YMC, Kyoto, Japan), and Sephadex LH-20 (Pharmacia Fine Chemicals, Uppsala, Sweden). Preparative HPLC separation was performed using a Shimadzu LC-10AT instrument with an SPD-10A detector using a YMC-Pack ODS-A column (250 mm × 20 mm, 5 *μ*m).

### Plant material

The roots of *Ranunculus ternatus* Thunb were purchased from Beijing Pu Sheng Lin Pharmaceutical Co., Ltd. in Beijing, China, in Feb 2010. The plant material was identified by Prof. Ma Lin (Institute of Materia Medica, Peking Union Medical College and Chinese Academy of Medical Sciences, Beijing 100050, P R. China). A specimen was deposited at the Herbarium of the Department of Medicinal Plants, Institute of Materia Medica, Beijing, China.

### Extraction and isolation

Air-dried powder roots of *Ranunculus ternatus* Thunb (20.0 kg) were extracted with 95% EtOH twice and then extracted with 70% EtOH once. The residue (5.1 kg) obtained by concentrating the EtOH extract in vacuo was suspended in water and extracted with petroleum ether, ethyl acetate and n-butanol. The remaining water portion after extraction was subjected to HP-20 macroporous resin using an H2O-EtOH gradient to give six fractions. The 30% ethanol elutant from the macroporous resin column was separated into two subfractions by Sephadex LH-20 using an H_2_O-MeOH gradient. Subsequently, subfraction 1 was processed by a combination of reversed phase silica gel, Sephadex LH-20 column chromatography and preparative HPLC to yield compounds **1** (15 mg), **2** (12 mg), and **3** (10 mg) and subfraction 2 to give **4** (8 mg). The purity of the isolated species were shown by HPLC (Figure S32-S35 in Supporting Information). The experiments were performed on an Agilent 1100 system using an apollo C18 column (250 × 20 mm, 5 *μ*m) maintained at 40 °C under a flow rate of 1 mL/min. The chromatograms of these compounds were achieved under 295 nm with a programmed gradient of methanol (A) and water-acetic acid (100:0.2, v/v. B): 0–30 min, 2–60% A; 30–40 min, 60–100% A.

Compound **1**, yellow amorphous powder, 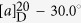 (c 0.206 MeOH). UVλ_max_ (MeOH): 295 nm. IR: 1712 cm^−1^, 1649 cm^−1^. ESI-MS m/z: 374.4 [*M* + H]^+^, HRESIMS: *m/z* 396.1266 [*M* + Na]^+^ (calcd for 396.1265). ^1^H, ^13^C NMR data (500 MHz, DMSO- *d*_6_), see Table 1.

Compound **2**, yellow amorphous powder, 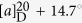 (c 0.12 MeOH). UVλ_max_ (MeOH): 295 nm. IR: 1708 cm^−1^, 1647 cm^−1^. ESI-MS m/z: 374.5 [*M* + H]^+^, HRESIMS: *m/z* 396.1266 [*M* + Na]^+^ (calcd for 396.1265). ^1^H, ^13^C NMR data (500 MHz, DMSO- *d*_6_), see Table 1.

Compound **3**, yellow amorphous powder, 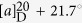 (c 0.12 MeOH). UVλ_max_ (MeOH): 295 nm. IR: 1706 cm^−1^, 1647 cm^−1^. ESI-MS m/z: 536.1. [*M* + H]^+^, HRESIMS: *m/z* 558.1791. [*M* + Na]^+^ (calcd for 558.1793). ^1^H, ^13^C NMR data (500 MHz, DMSO- *d*_6_), see Table 2.

Compound **4**, yellow amorphous powder, 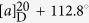 (c 0.054 MeOH). UVλ_max_ (MeOH): 298 nm. IR: 1776 cm^−1^, 1712 cm^−1^, 1650 cm^−1^. ESI-MS m/z: [*M* + H]^+^ 488.1, HRESIMS: *m/z* 510.1587 [*M* + Na]^+^ (calcd for 510.1582). ^1^H, ^13^C NMR data (500 MHz, DMSO-*d*_6_), see Table 2.

## Additional Information

**How to cite this article**: Feng, Z.-m. *et al.* Naturally occurring hybrids derived from γ-amino acids and sugars with potential *tail to tail* ether-bonds. *Sci. Rep.* **6**, 25443; doi: 10.1038/srep25443 (2016).

## Supplementary Material

Supplementary Information

## Figures and Tables

**Figure 1:**
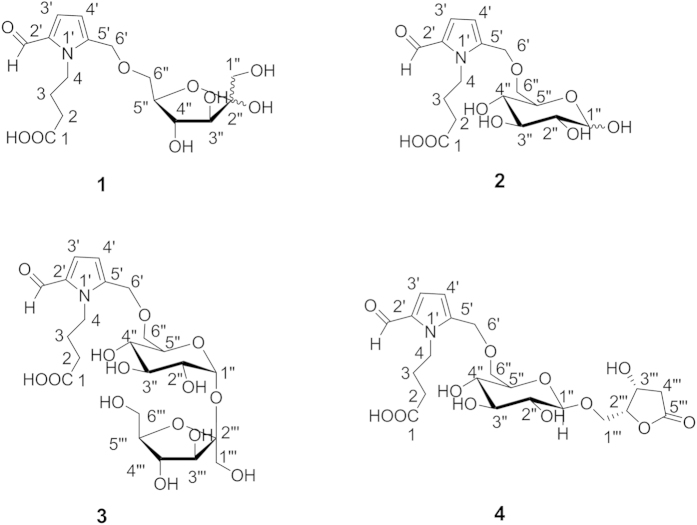
The structures of compounds 1–4.

**Table 1:** NMR data of compounds **1–2** at 500 MHz in DMSO-*d*_6_.

1					2			
Position	*δ*_H_ 1*α	*δ*_*C*_1*α	*δ*_H_ 1*β	*δ*_*C*_1*β	*δ*_H_ 2*α	*δ*_*C*_2*α	*δ*_H_ 2*β	*δ*_*C*_2*β
CHO	9.48 s	179.4	9.48 s	179.4	9.48 s	179.4	9.48 s	179.4
1		173.8		173.9		174.1		174.1
2	4.25 t (7.5)	44.2	4.25 t (7.5)	44.2	4.25 m	44.3	4.25 m	44.3
3	1.85 t (7.5)	26.3	1.85 t (7.5)	26.3	1.85 m	26.4	1.85 m	26.4
4	2.20 t (7.5)	30.7	2.20 t (7.5)	30.7	2.20 m	30.9	2.20 m	30.9
2′		132.0		132.0		131.9		131.9
3′	6.98 d (3.5)	123.6	6.98 d (3.5)	123.6	6.98 d (3.5)	123.5	6.98 d (3.5)	123.6
4′	6.27 d (3.5)	111.2	6.27 d (3.5)	111.1	6.25 d (3.5)	111.1	6.25 d (3.5)	111.2
5′		139.2		139.1		139.3		139.2
6′	4.50 s	63.6	4.50 s	63.3	4.50 s	63.5	4.50 s	63.4
1″	3.38 m,3.30 m	63.4	3.22 dd (17.0, 12.0)	62.9	4.88 d (3.5)	92.2	4.27 m	96.8
2″		104.4		102.2	3.71 m	70.4	2.87 t (8.5)	74.7
3″	3.82 d (7.5)	79.2	3.82 d (7.5)	75.2	3.10 m	72.2	3.25 m	74.9
4″	3.58 m	77.2	3.75 t (7.5)	75.7	2.98 m	70.6	2.98 m	70.3
5″	3.78 m	82.9	3.63 m	79.6	3.40 m	73.1	3.09 m	76.6
6″	3.44 m, 3.43 m	70.8	3.57 m, 3.45 m	72.4	3.64 m, 3.49 m	70.0	3.70 m, 3.46 m	69.9

**Table 2:** NMR data of compound **3–4** at 500 MHz in DMSO-*d*_6_.

Position	3		4	
	*δ*_H_	*δ*_C_	*δ*_H_	*δ*_C_
CHO	9.48 s	179.4	9.47 s	179.4
1		174.0		173.9
2	2.20 t (7.5)	30.9	2.14 t (7.0)	31.4
3	1.85 m	26.4	1.85 m	26.7
4	4.25 m	44.2	4.25 t (7.0)	44.4
2′		131.9		132.0
3′	6.98 d (3.5)	123.6	6.98 d (4.0)	123.6
4′	6.27 d (3.5)	111.2	6.27 d (4.0)	111.2
5′		139.3		139.2
6′	4.58 d (17.5), 4.48 d (17.5)	63.3	4.50 s	63.7
1″	5.12 d (3.5)	91.8	4.23 d (8.0)	103.0
2″	3.18 dd (10.0, 3.5)	71.7	2.95 t (9.0)	73.3
3″	3.48 m	72.8	3.12 t (9.0)	76.6
4″	3.07 t (9.5)	70.2	3.03 t (9.0)	69.9
5″	3.64 m	72.9	3.30 m	75.3
6″	3.78 m, 3.56 d (9.0)	71.9	3.73 d (6.0), 3.52 dd (11.0, 6.0)	69.6
1″′	3.39 s	61.8	4.00 m, 3.68 dd (12.0, 8.0)	67.6
2″′		104.1	4.52 m	83.2
3″′	3.71 m	76.9	4.39 m	67.1
4″′	3.86 m	75.1	2.82 dd (17.0, 5.5), 2.25 d (17.0)	38.8
5″′	3.69 m	80.5		175.9
6″′	3.62 m,3.49 m	61.0		
