# Supplementary material for: Naturally occurring hybrids derived from γ-amino acids and sugars with potential tail to tail ether-bonds
Source: Sci Rep. 2016 May 11;6:25443. doi: 10.1038/srep25443 (PMC4863253; doi:10.1038/srep25443)
Supplement: Supplementary Information [file srep25443-s1.pdf]

Supplementary Information

Naturally occurring hybrids derived from γ-amino acids and sugars with potential *tail to tail* ether-bonds

Zi-ming Feng [1,⊥], Zhi-lai Zhan [1,2,⊥], Ya-nan Yang [1], Jian-shuang Jiang [1], Pei-cheng Zhang [1,*]

[1] State Key Laboratory of Bioactive Substance and Function of Natural Medicines, Institute of Materia Medica, Chinese Academy of Medical Sciences and Peking Union Medical College, Beijing 100050, P. R. China

[2] State Key Laboratory Breeding Base of Dao-di Herbs，National Resource Center for Chinese Materia Medica, China Academy of Chinese Medical Sciences, Beijing, 100700, P. R. China

[*] To whom correspondence should be addressed. Tel: 86-10-63165231. Fax: 86-10-63017757. E-mail: pczhang@imm.ac.cn

⊥ These authors contributed equally to the work.

SUPPORTING FIGURES

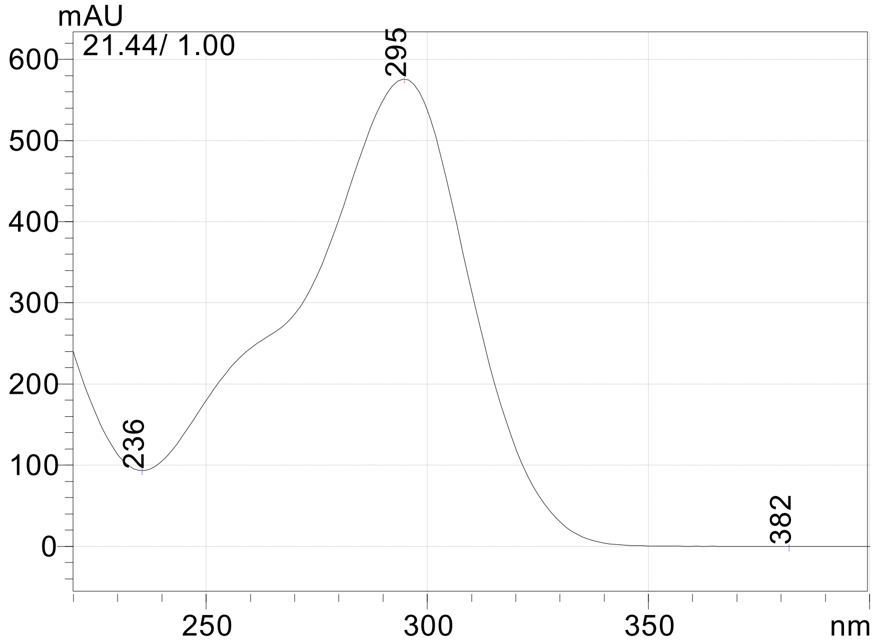

Figure S1. UV spectrum of compound **1**

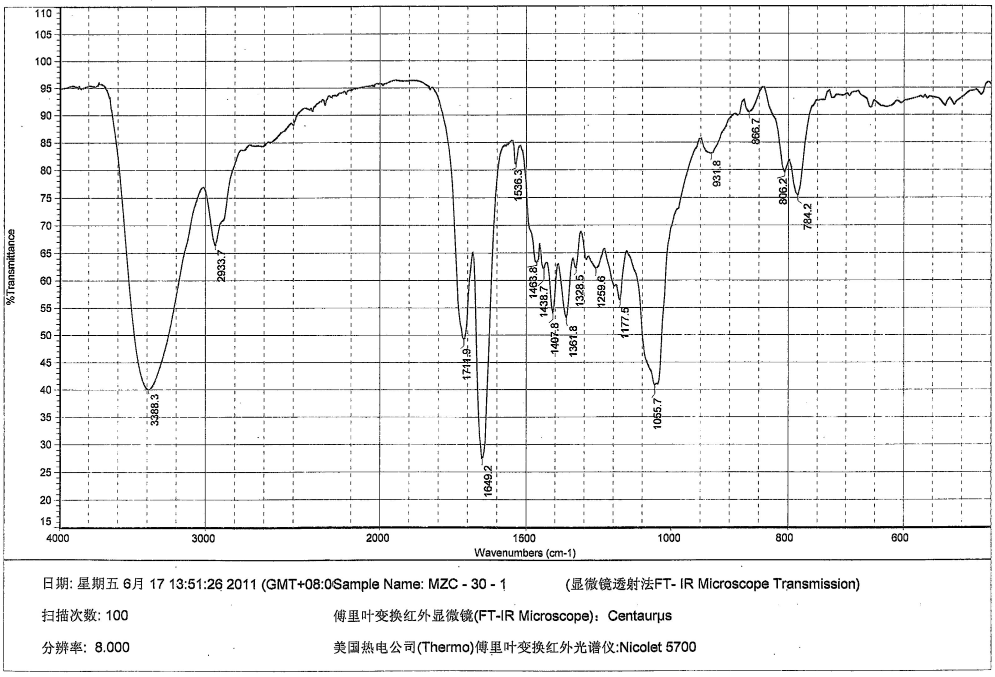

Figure S2. The IR spectrum of **1**

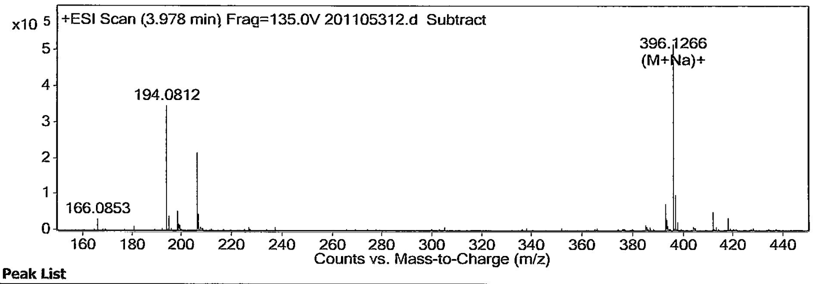

Peak List

m/z	z	Abund	Formula	Ion
194.0812	1	347618		
198.5608		53984		
206.5496	2	216328		
207.0511	2	43485		
393.1191	2	72272		
396.1266	1	517116	C16 H23 N Na O9	(M+Na)+
397.1287	1	99108	C16 H23 N Na O9	(M+Na)+
412.0949		51026		
769.256		58758		
785.2282		70046		

Formula Calculator Element Limits

Element	Min	Max
C	3	100
H	0	120
O	0	30
N	0	5
S	0	2
Cl	0	0

Formula Calculator Results

Formula	Best	Mass	Tgt Mass	Diff (ppm)	Ion Species	Score
C16 H23 N O9	TRUE	373.1374	373.1373	-0.21	C16 H23 N Na O9	99.77
C17 H19 N5 O5		373.1374	373.1386	3.37	C17 H19 N5 Na O5	99.51
C21 H19 N5 S		373.1374	373.1361	-3.34	C21 H19 N5 Na S	98.61
C20 H23 N O4 S		373.1374	373.1348	-6.92	C20 H23 N Na O4 S	98.57
C17 H27 N O4 S2		373.1374	373.1381	2.11	C17 H27 N Na O4 S2	97.98
C13 H27 N O9 S		373.1374	373.1407	8.82	C13 H27 N Na O9 S	97.96
C18 H23 N5 S2		373.1374	373.1395	5.69	C18 H23 N5 Na S2	97.7
C12 H27 N3 O6 S2		373.1374	373.1341	-8.68	C12 H27 N3 Na O6 S2	96.45

Figure S3. The HR-ESI-MS of **1**

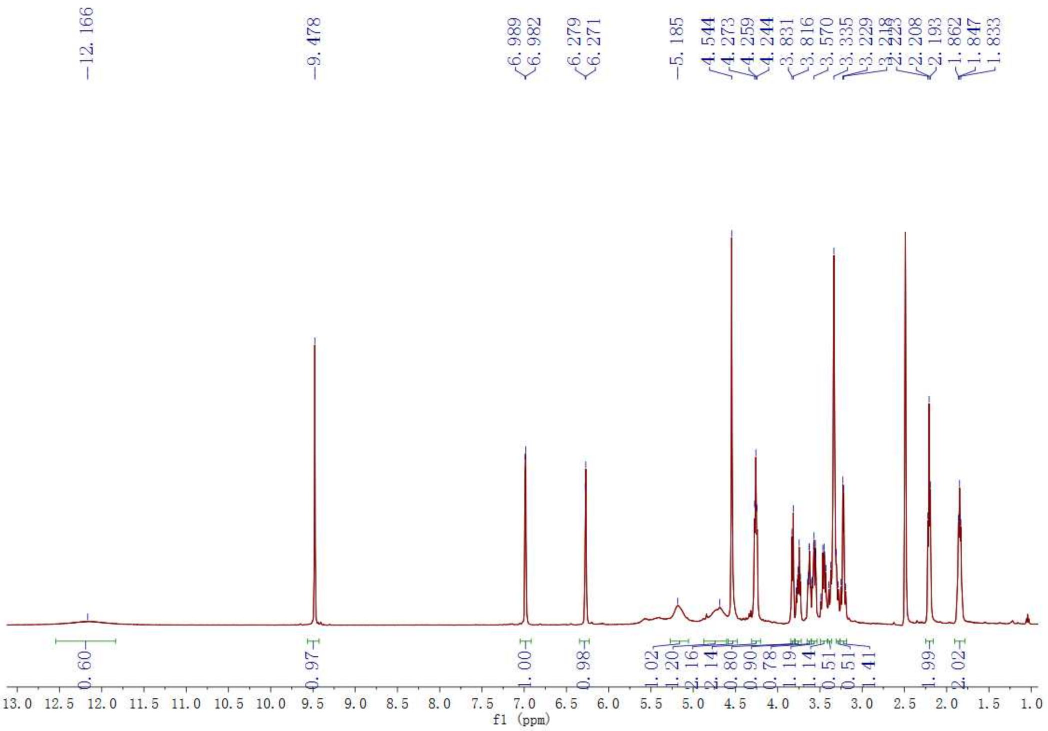

Figure S4. The ^{1}H NMR spectrum of **1** in DMSO-d_6.

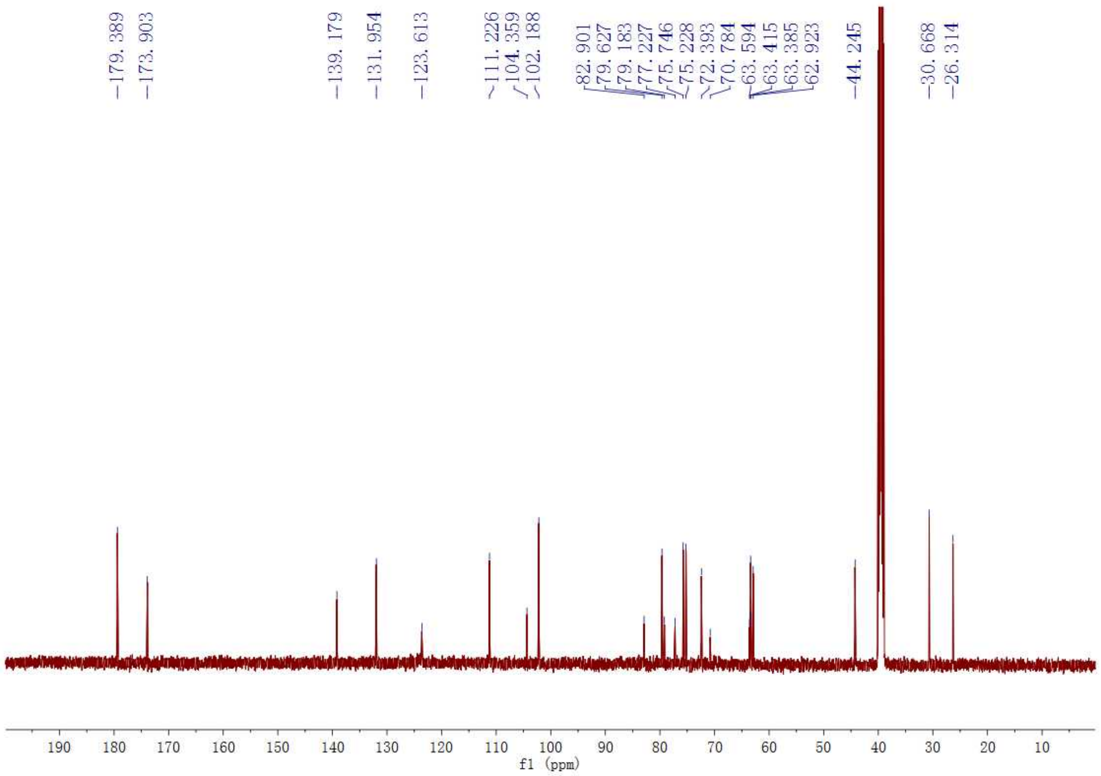

Figure S5. The ^{13}C NMR spectrum of **1** in DMSO-d_6.

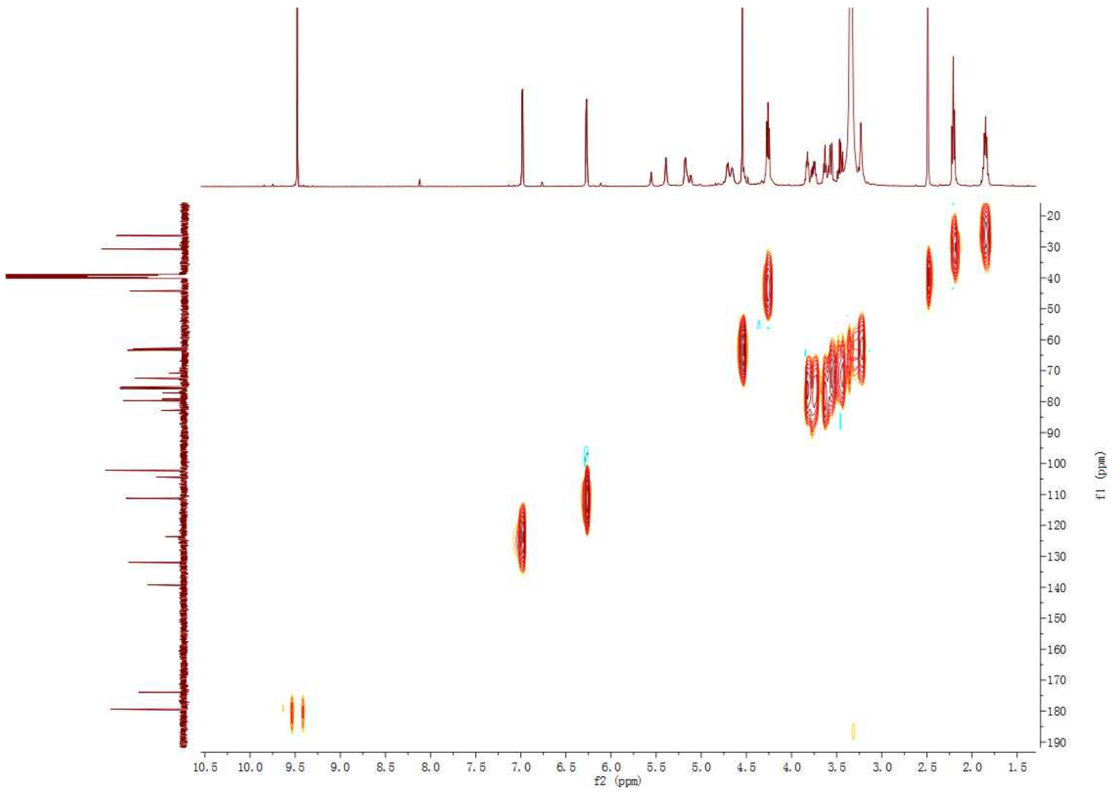

Figure S6. The HSQC spectrum of **1** in DMSO-d_6.

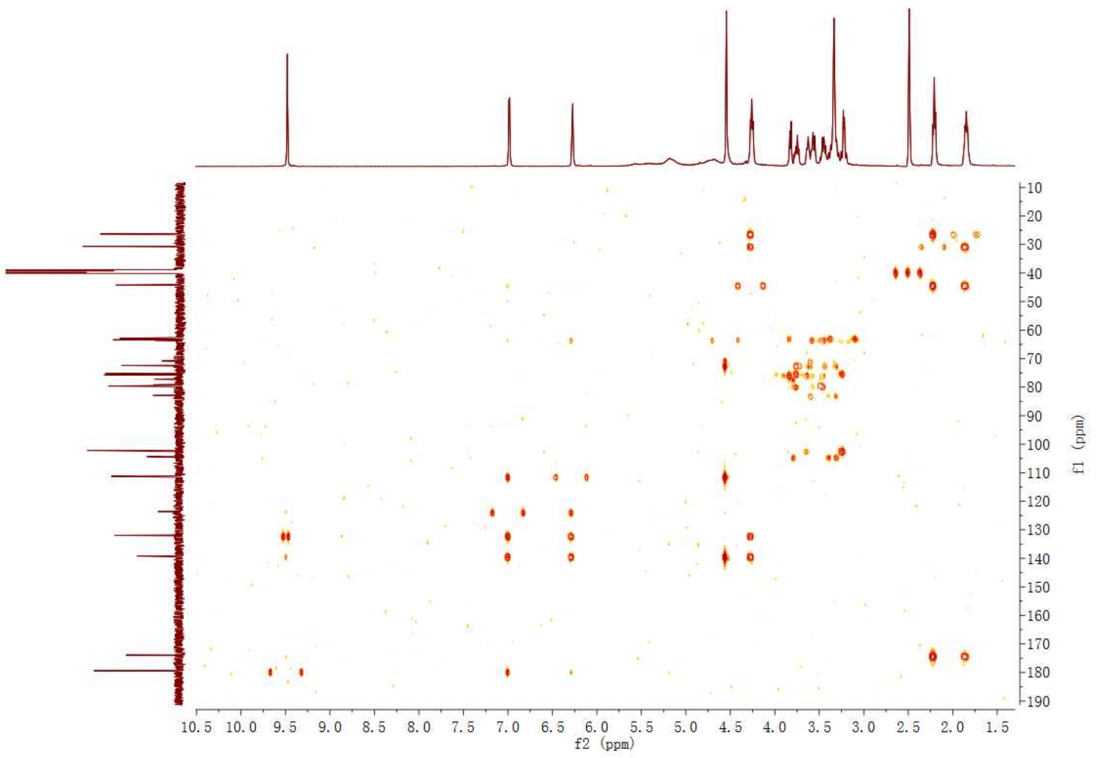

Figure S7. The HMBC spectrum of **1** in DMSO-d_6.

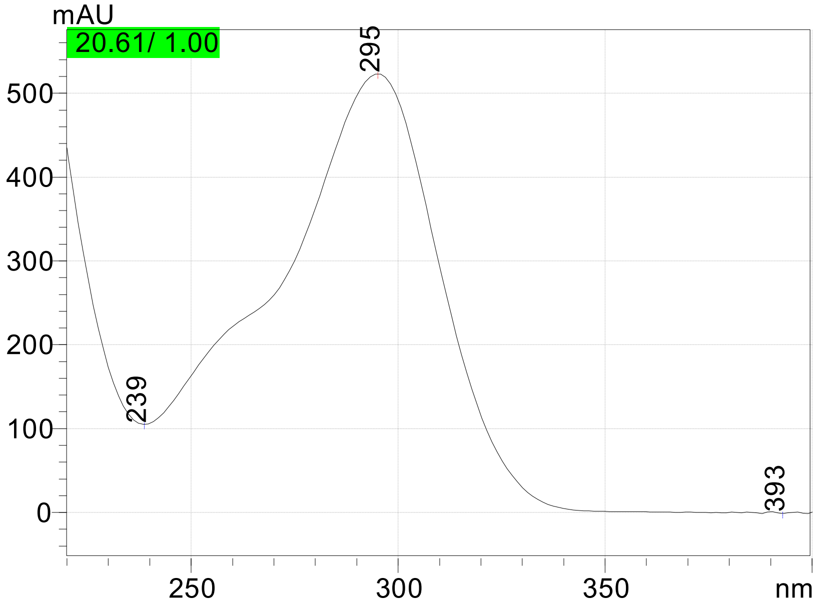

Figure S8. UV spectrum of compound **2**

Qualitative Analysis Report

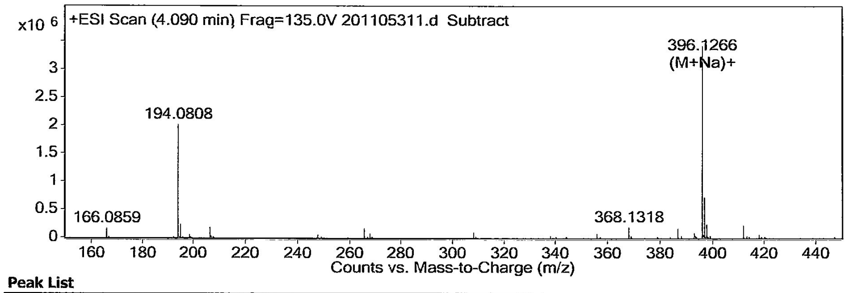

Peak List

m/z	z	Abund	Formula	Ion
194.0808	1	2023160		
195.0843	1	245973		
396.1266	1	3415181	C16 H23 N Na O9	(M+Na)+
396.2954		217325		
397.1305	1	729069	C16 H23 N Na O9	(M+Na)+
398.1367	1	236422	C16 H23 N Na O9	(M+Na)+
412.0981		220534		
769.2532		242756		
785.2291	1	531013		
791.2438		294927		

Formula Calculator Element Limits

Element	Min	Max
C	3	100
H	0	120
O	0	30
N	0	5
S	0	2
Cl	0	0

Formula Calculator Results

Formula	Best	Mass	Tgt Mass	Diff (ppm)	Ion Species	Score
C16 H23 N O9	TRUE	373.1374	373.1373	-0.39	C16 H23 N Na O9	99.15
C17 H19 N5 O5		373.1374	373.1386	3.18	C17 H19 N5 Na O5	98.99
C21 H19 N5 S		373.1374	373.1361	-3.53	C21 H19 N5 Na S	98.46
C20 H23 N O4 S		373.1374	373.1348	-7.11	C20 H23 N Na O4 S	98.35
C17 H27 N O4 S2		373.1374	373.1381	1.92	C17 H27 N Na O4 S2	97.82
C18 H23 N5 S2		373.1374	373.1395	5.49	C18 H23 N5 Na S2	97.6
C13 H27 N O9 S		373.1374	373.1407	8.63	C13 H27 N Na O9 S	97
C12 H27 N3 O6 S2		373.1374	373.1341	-8.87	C12 H27 N3 Na O6 S2	95.66

--- End Of Report ---

Figure S9. The HR-ESI-MS of **2**

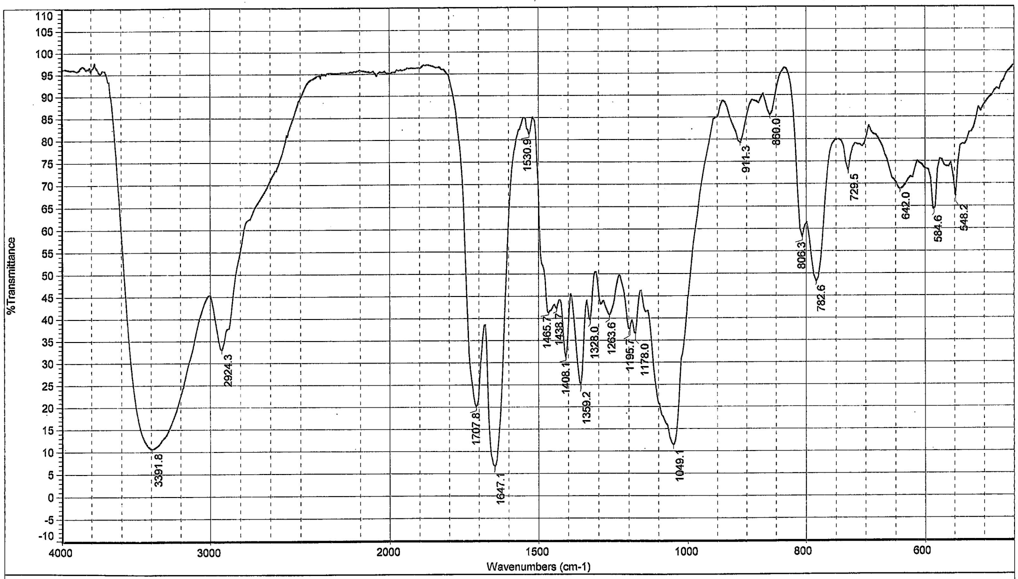

日期: 星期五 6月 17 13:58:31 2011 (GMT+08:0Sample Name: MZC - 30 - 2 (显微镜透射法FT- IR Microscope Transmission)

扫描次数: 100 傅里叶变换红外显微镜(FT-IR Microscope)：Centaurµs

分辨率: 8.000 美国热电公司(Thermo)傅里叶变换红外光谱仪:Nicolet 5700

Figure S10. The IR spectrum of **2**

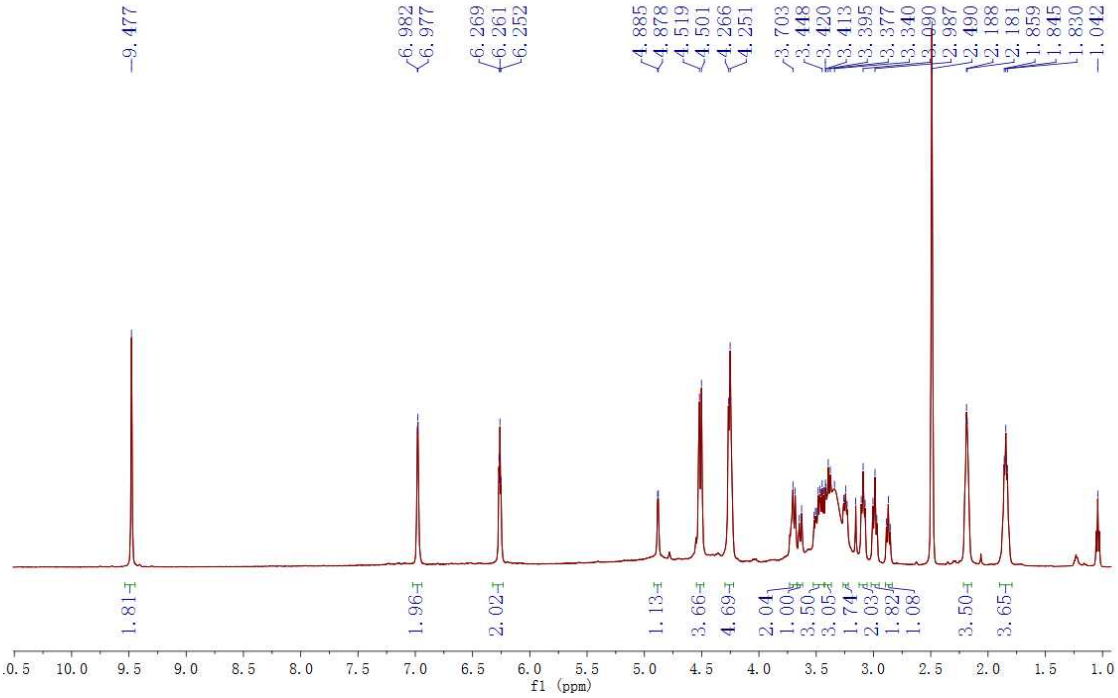

Figure S11. The ^{1}H NMR spectrum of **2** in DMSO-d_6.

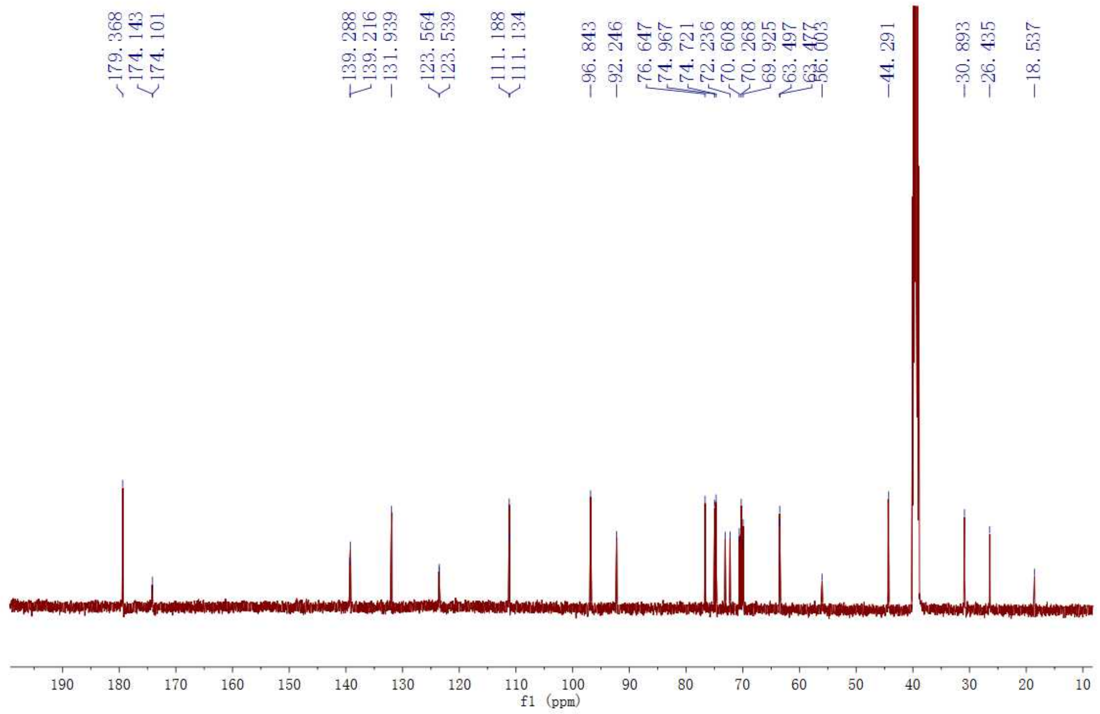

Figure S12. The ^{13}C NMR spectrum of **2** in DMSO-d_6.

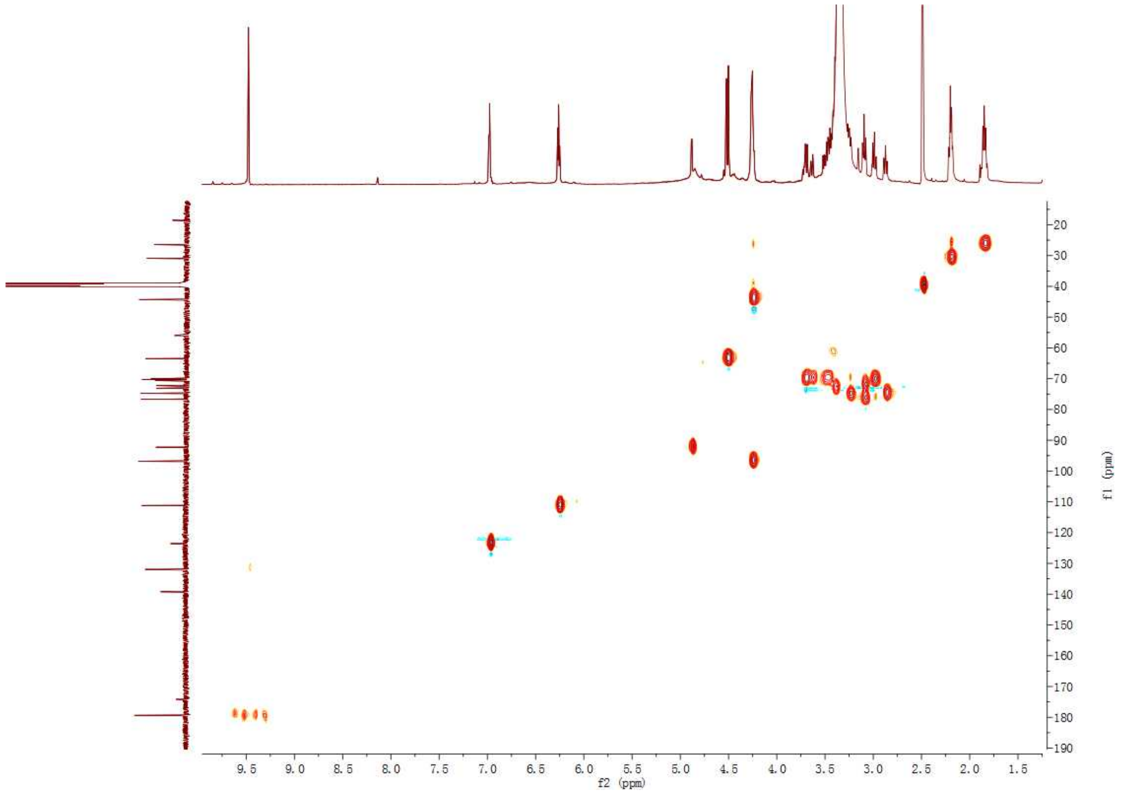

Figure S13. The HSQC spectrum of **2** in DMSO-d_6.

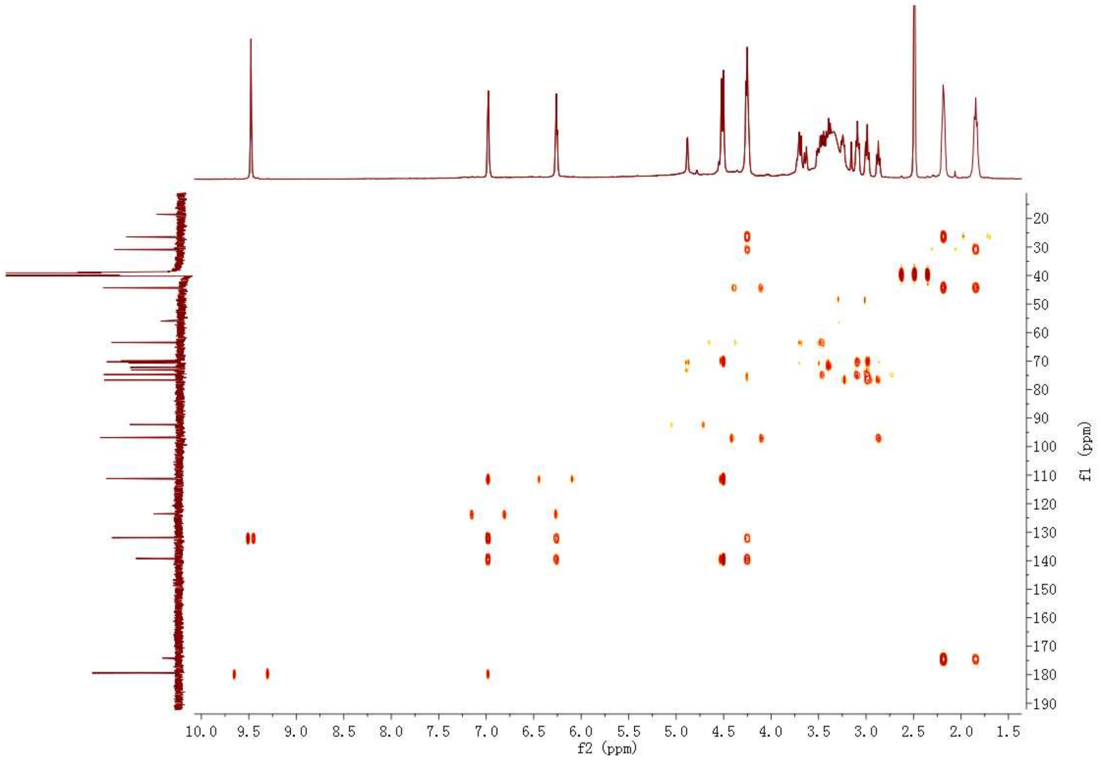

Figure S14. The HMBC spectrum of **2** in DMSO-d_6.

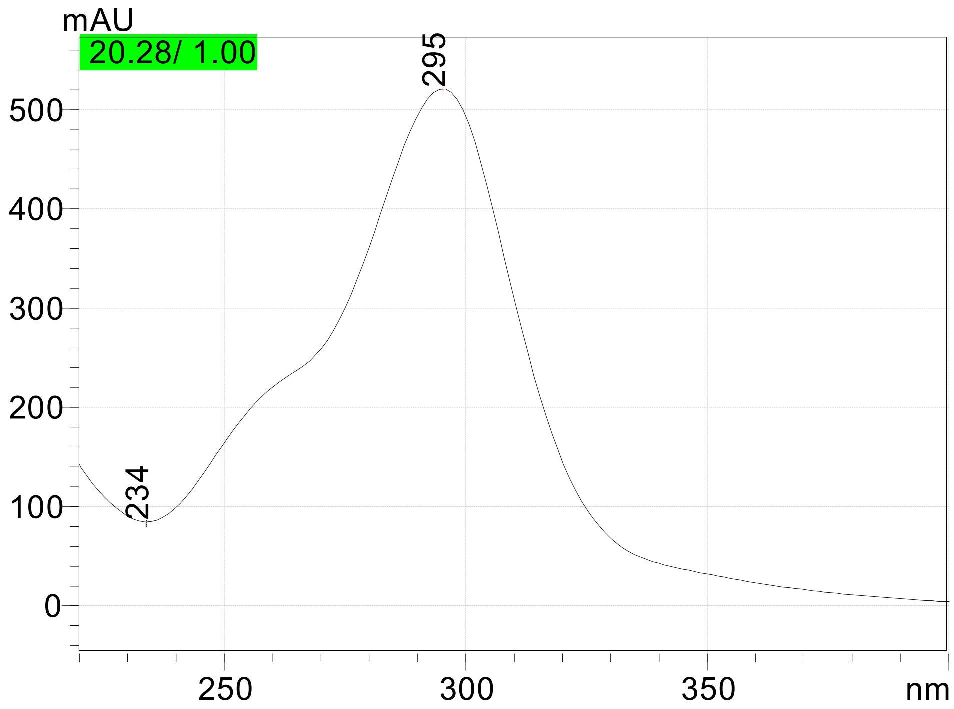

Figure S15. UV spectrum of compound **3**

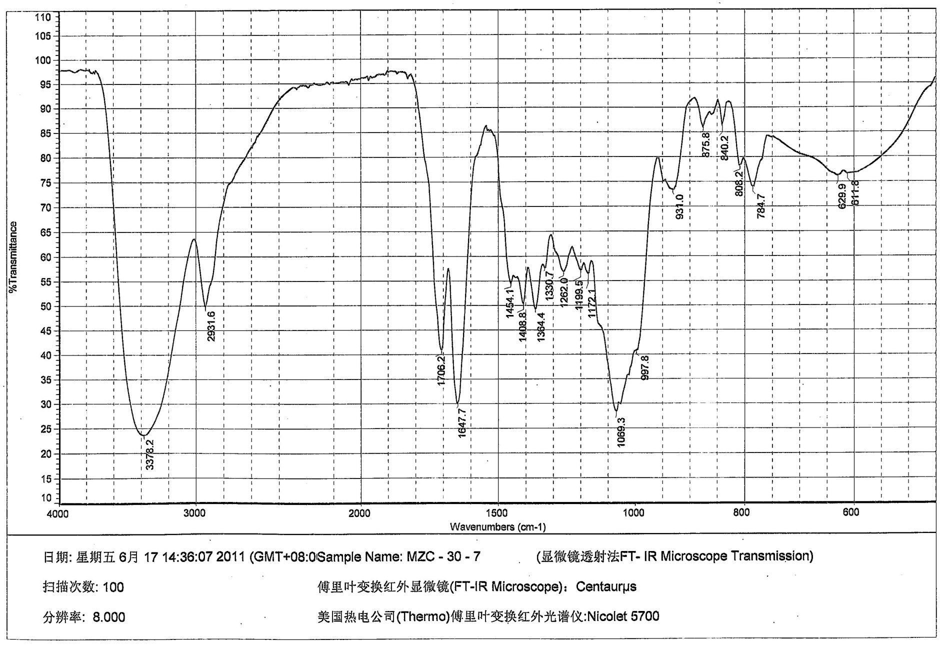

Figure S16. The IR spectrum of **3**

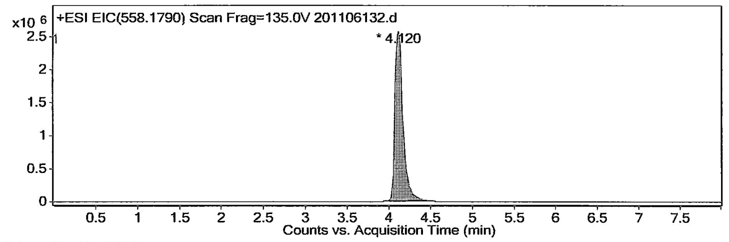

Integration Peak List

Peak	Start	RT	End	Height	Area	Area %
1	3.991	4.12	4.457	2569046	16867548	100

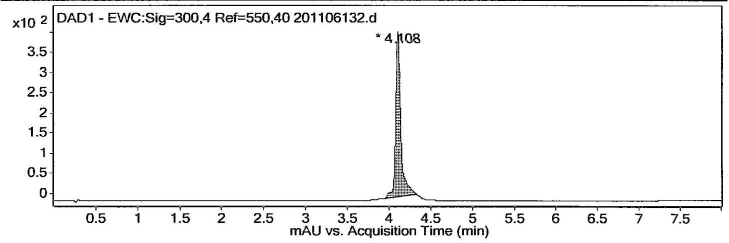

Integration Peak List

Peak	Start	RT	End	Height	Area	Area %
1	3.955	4.108	4.333	410.16	1690.509	100

User Spectra

Fragmentor Voltage	Collision Energy	Ionization Mode
135	0	Esi

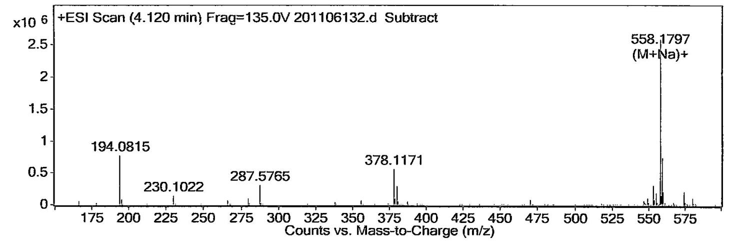

Figure S17. The HR-ESI-MS of **3**

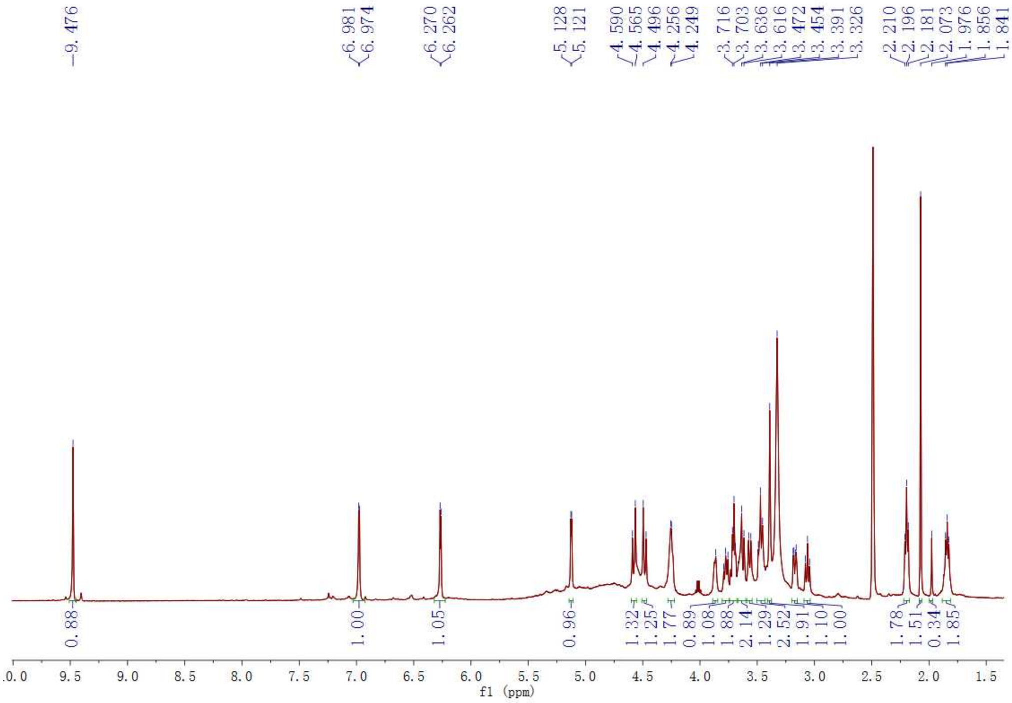

Figure S18. The ^{1}H NMR spectrum of **3** in DMSO-d_6.

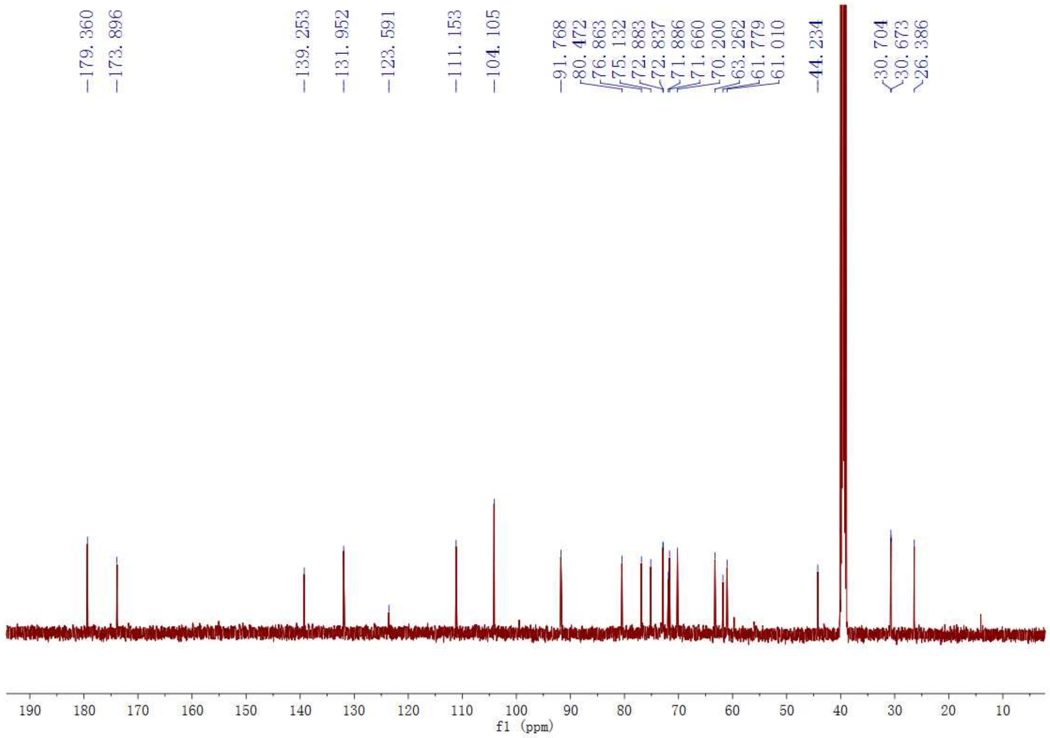

Figure S19. The ^{13}C NMR spectrum of **3** in DMSO-d_6.

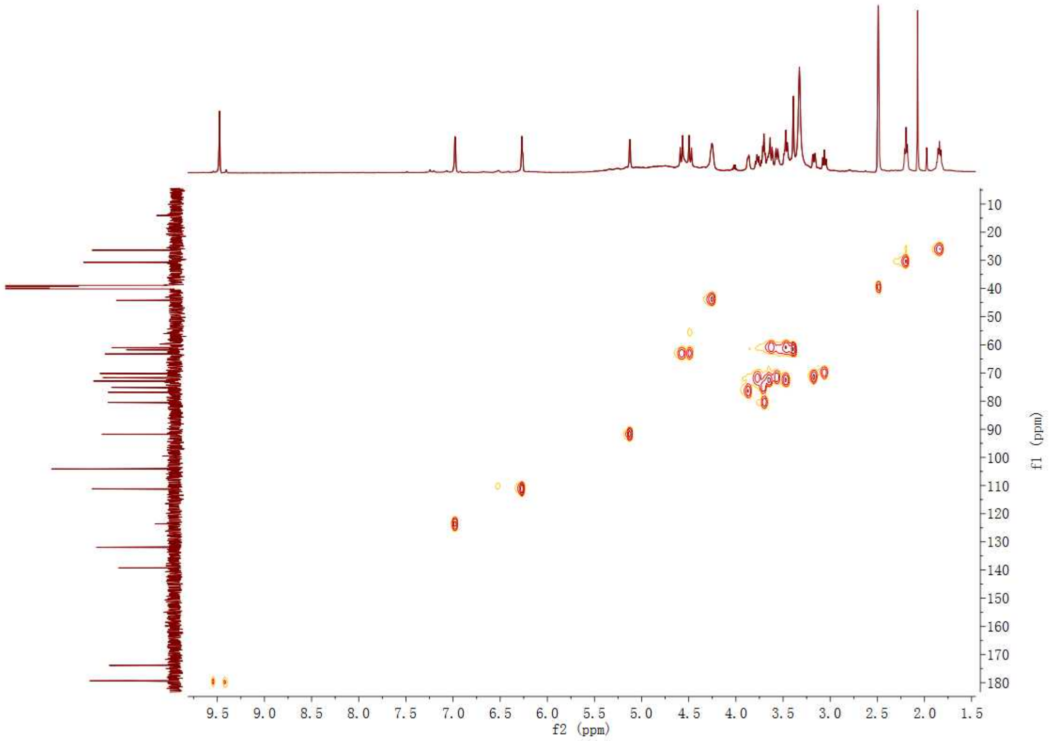

Figure S20. The HSQC spectrum of **3** in DMSO-d_6.

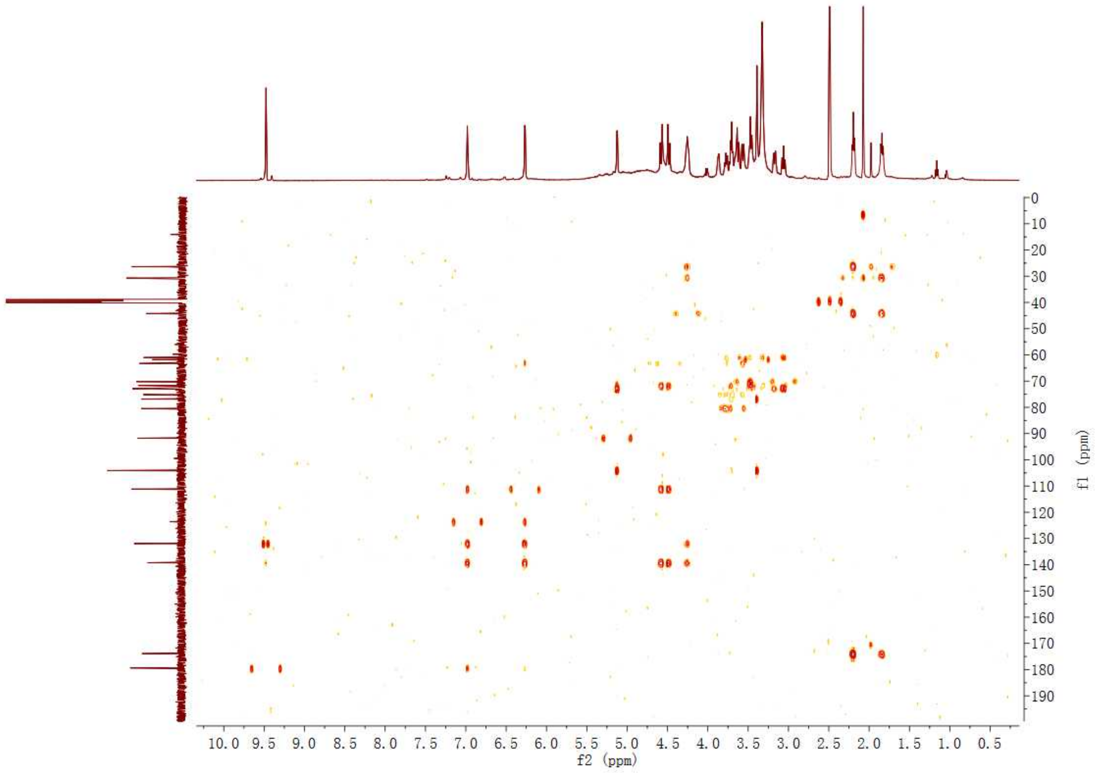

Figure S21. The HMBC spectrum of **3** in DMSO-d_6.

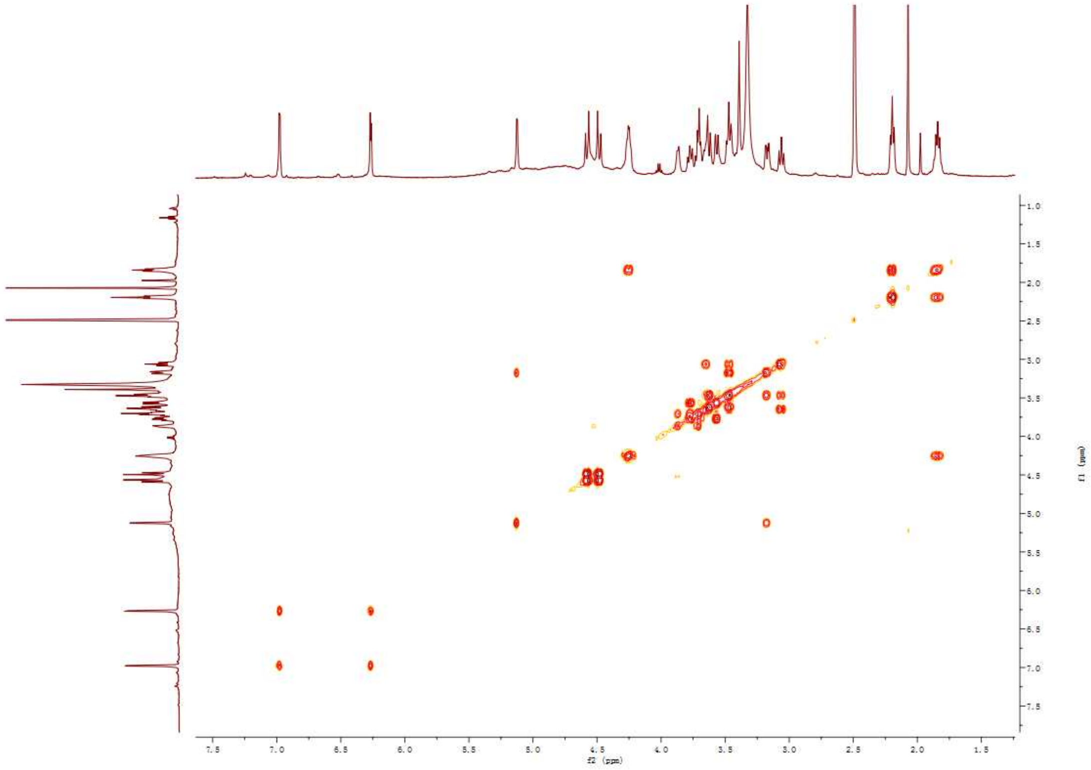

Figure S22. The ^{1}H-^{1}H COSY spectrum of **3** in DMSO-d_6.

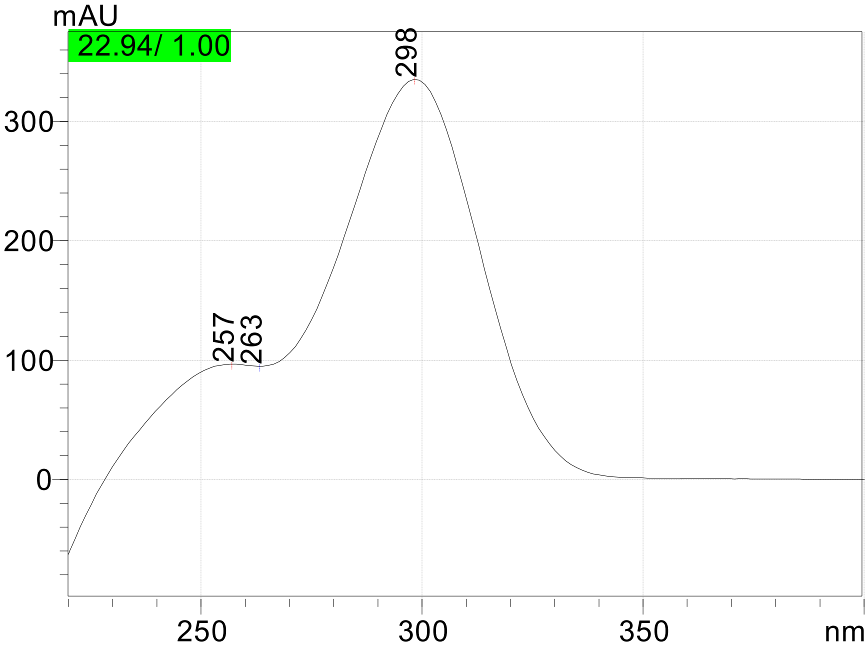

Figure S23. UV spectrum of compound **4**

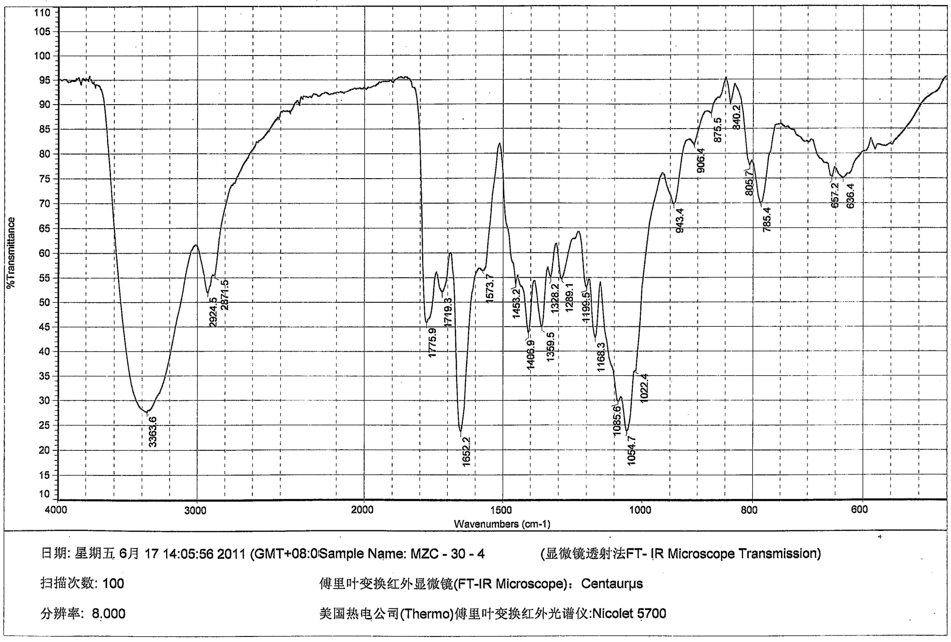

Figure S24. The IR spectrum of **4**

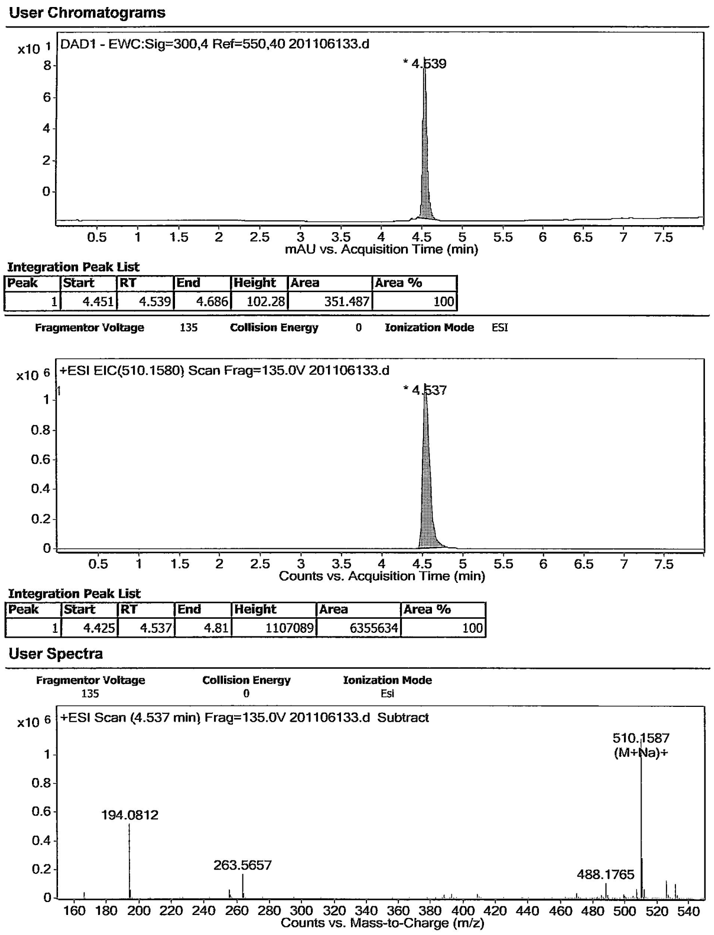

Figure S25. The HR-ESI-MS of **4**

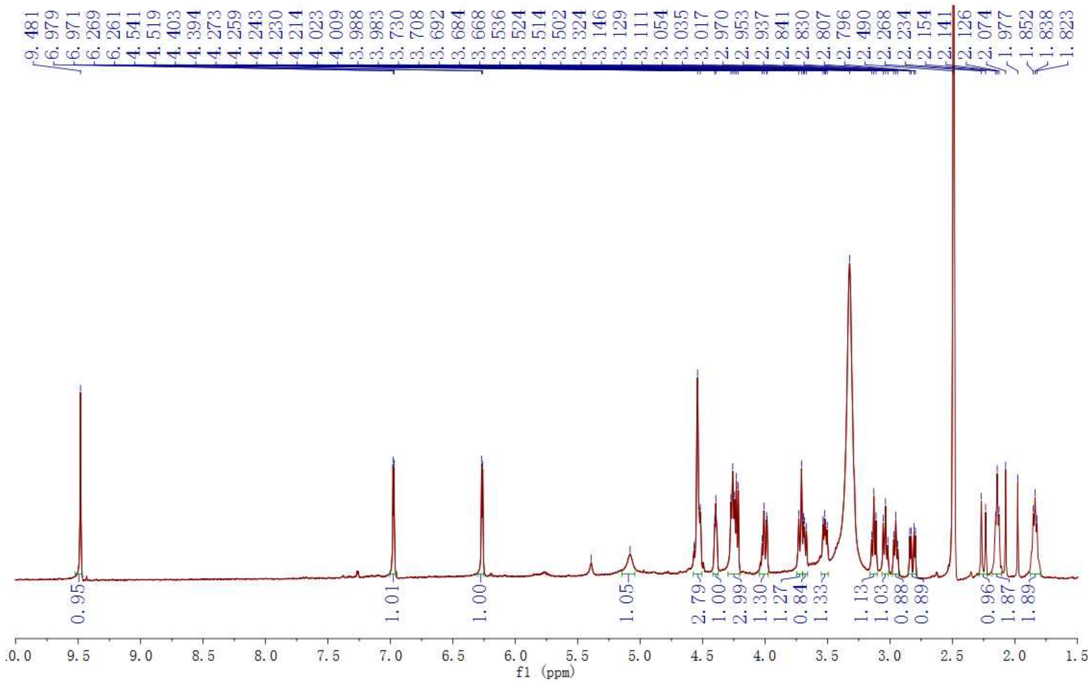

Figure S26. The ^{1}H NMR spectrum of **4** in DMSO-d_6.

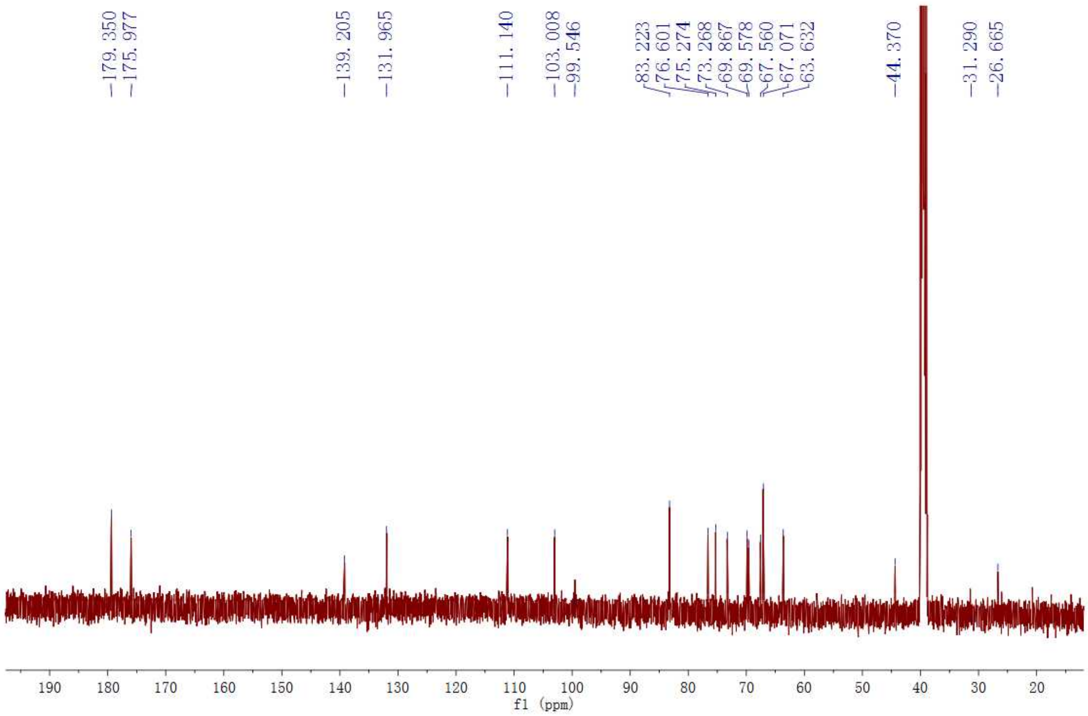

Figure S27. The ^{13}C NMR spectrum of **4** in DMSO-d_6.

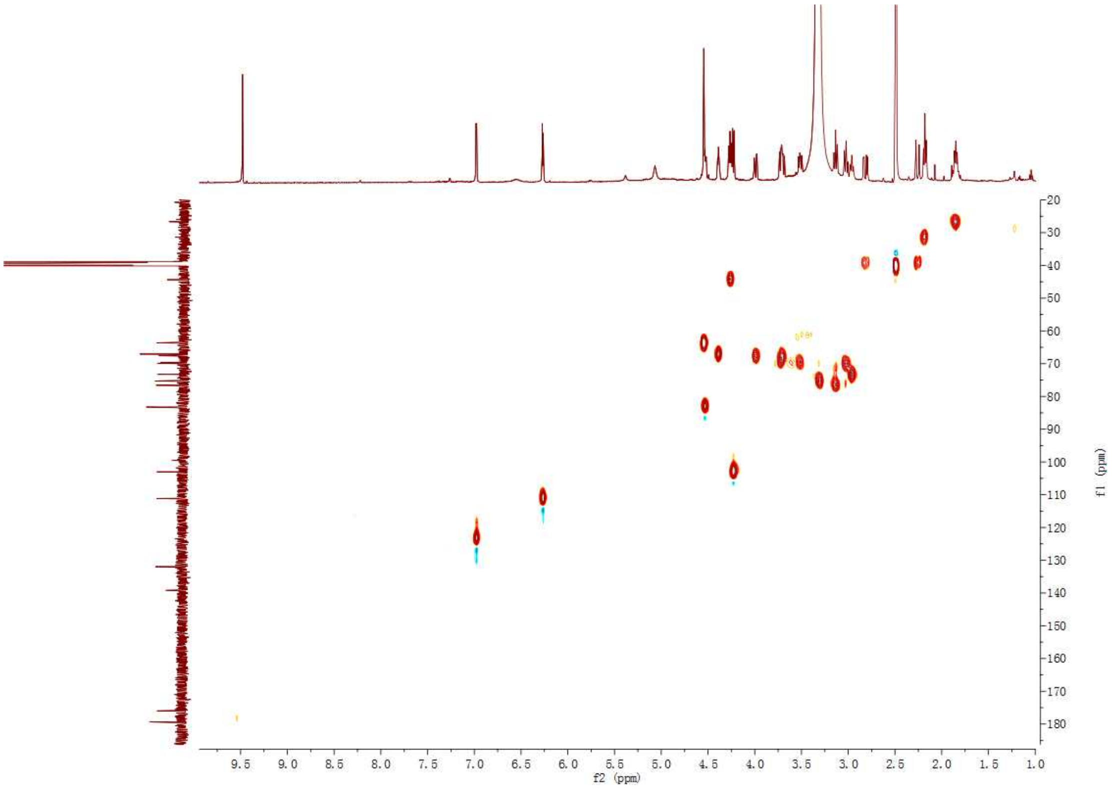

Figure S28. The HSQC spectrum of **4** in DMSO-d_6.

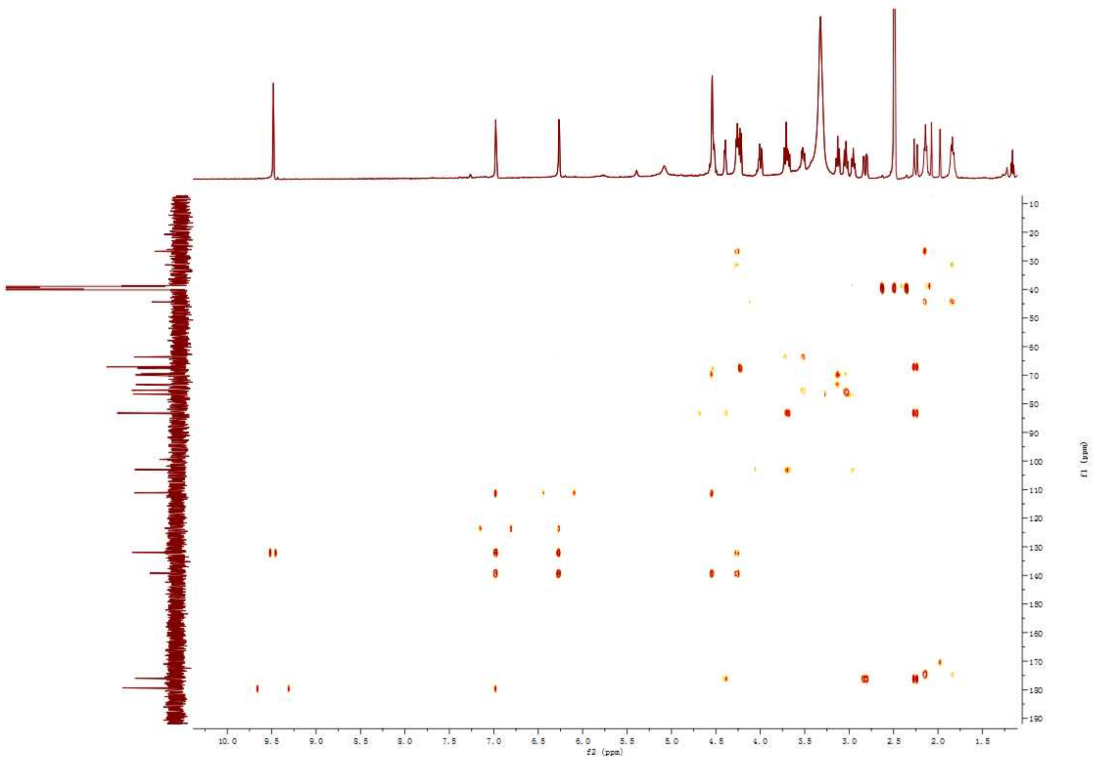

Figure S29. The HMBC spectrum of **4** in DMSO-d_6.

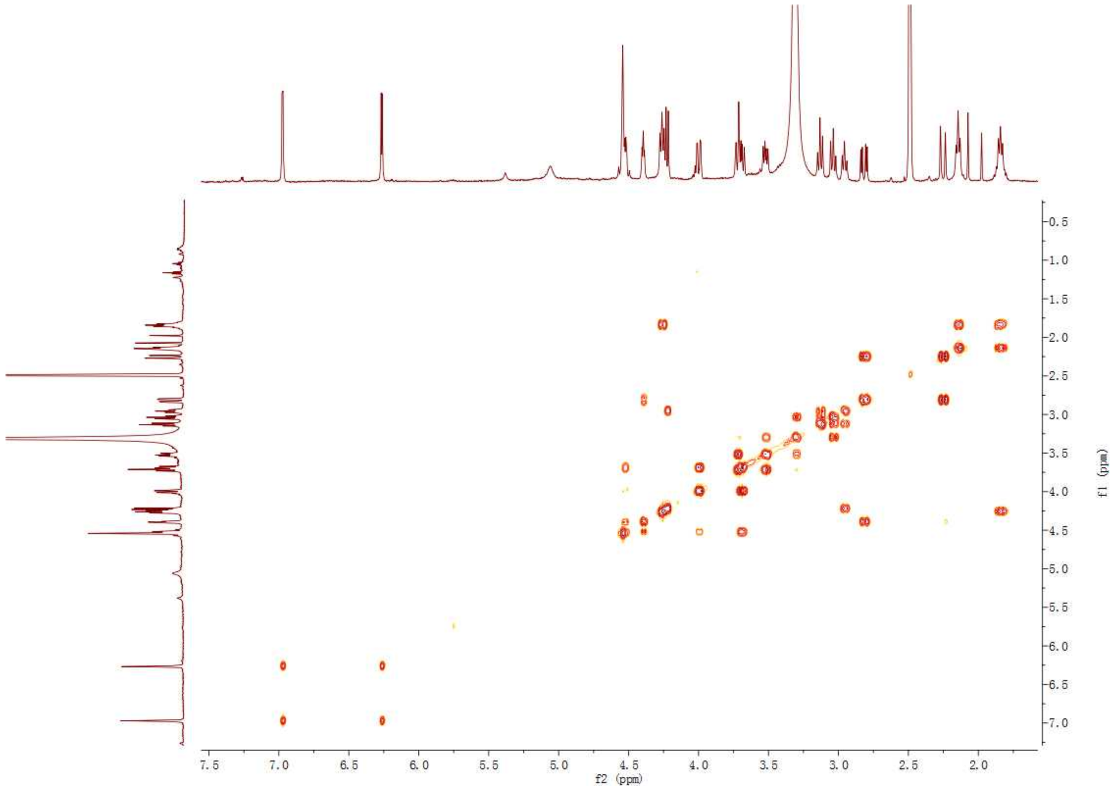

Figure S30. The ^{1}H-^{1}H COSY spectrum of **4** in DMSO-d_6.

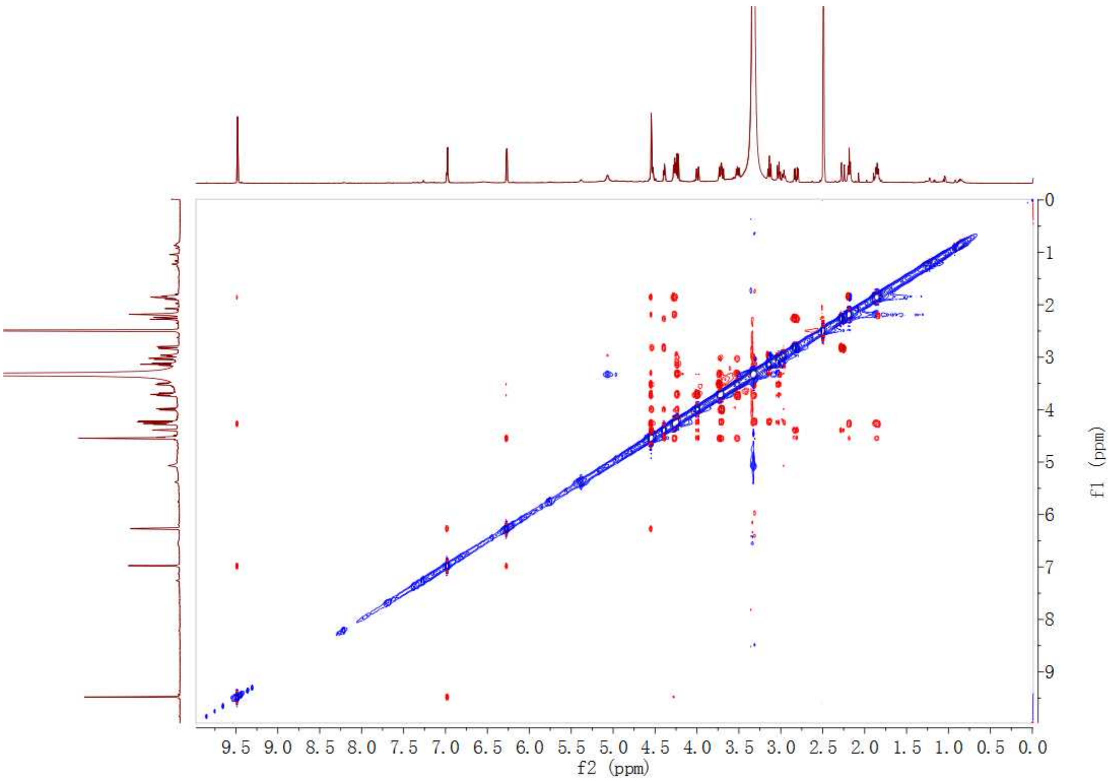

Figure S31. The ROESY spectrum of **4** in DMSO-d_6.

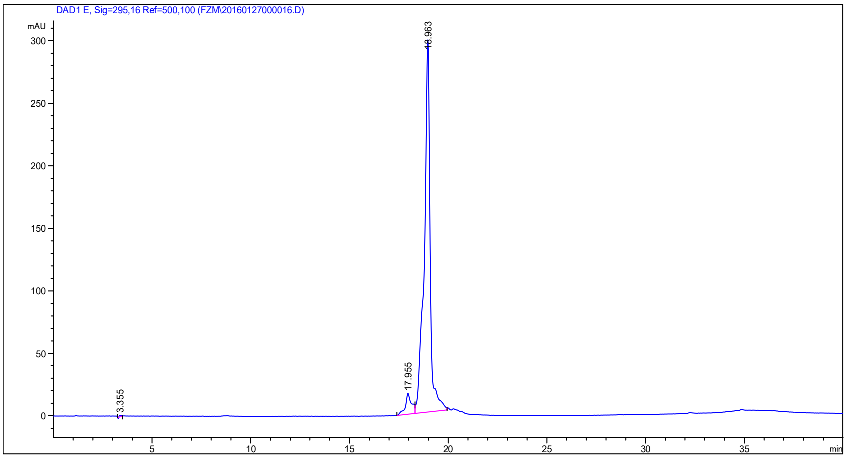

Figure S32. The chromatogram of compound **1** (**1***α* and **1***β* form)

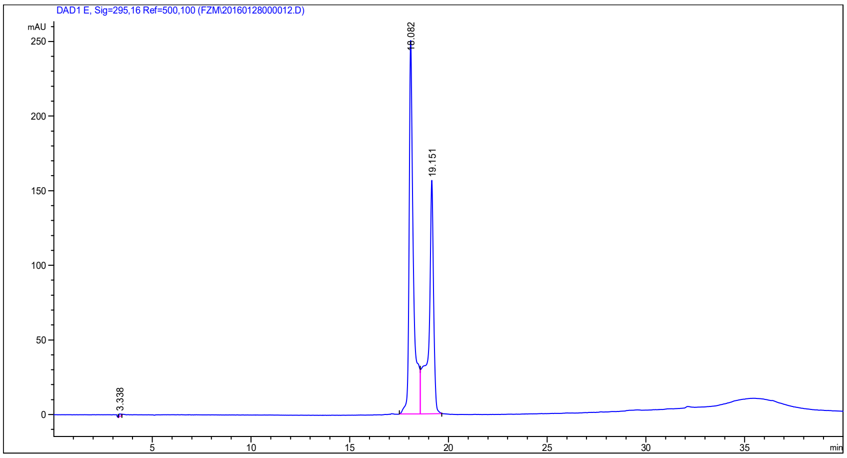

Figure S33. The chromatogram of compound **2** (**2***α* and **2***β* form)

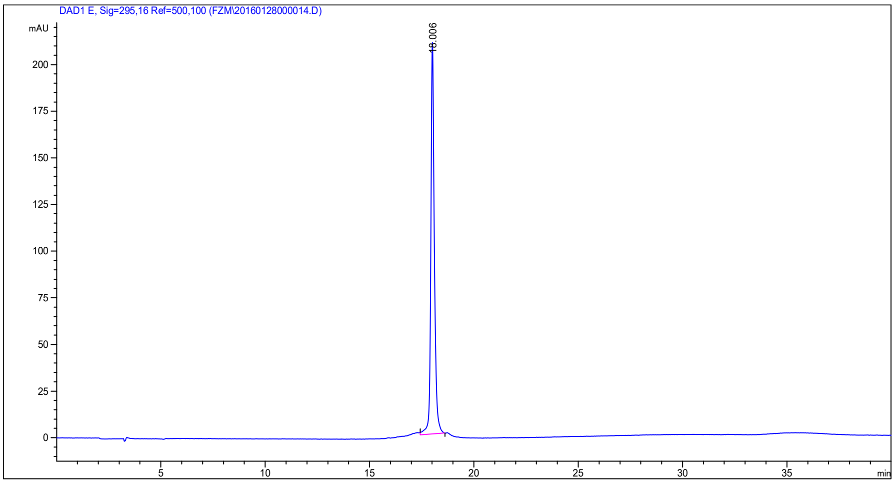

Figure S34. The chromatogram of compound **3**

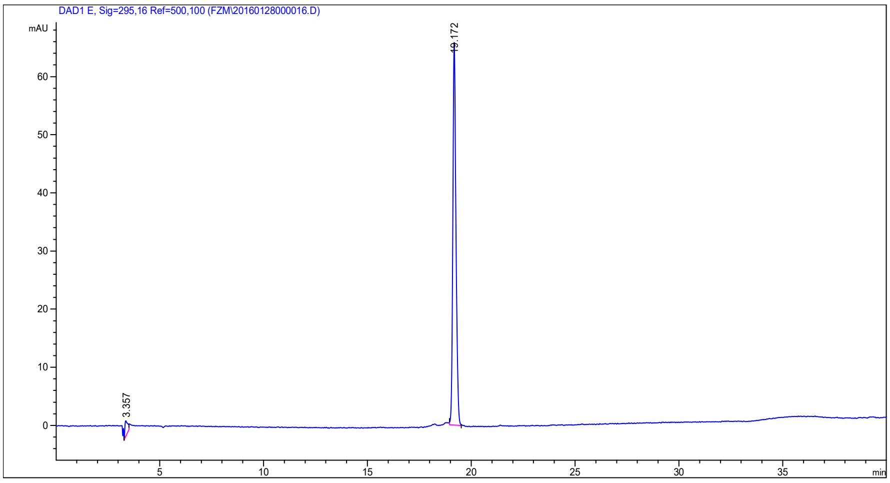

Figure S35. The chromatogram of compound **4**
